# Spatial immunogenomic patterns associated with lymph node metastasis in lung adenocarcinoma

**DOI:** 10.1186/s40164-024-00574-8

**Published:** 2024-10-28

**Authors:** Fanjie Meng, Hao Li, Ruoyi Jin, Airong Yang, Hao Luo, Xiao Li, Peiyu Wang, Yaxing Zhao, Olga Chervova, Kaicheng Tang, Sida Cheng, Bin Hu, Yun Li, Jianpeng Sheng, Fan Yang, David Carbone, Kezhong Chen, Jun Wang

**Affiliations:** 1 Department of Thoracic Surgery, Beijing Institute of Respiratory Medicine and Beijing Chao Yang Hospital, Capital Medical University, Beijing, China; 2 Department of Thoracic Surgery, Institution of Thoracic Oncology, Peking University People’s Hospital, No.11 Xizhimen South Street, Beijing, 100044 Xicheng District China; 3 Thoracic Oncology Institute & Research Unit of Intelligence Diagnosis and Treatment in Early Non-Small Cell Lung Cancer, Peking University People’s Hospital, Beijing, China; 4 Institute of Advanced Clinical Medicine, Peking University, Beijing, China; 5 Kanghui Biotechnology Co., Ltd, Shenyang, China; 6 Cancer Center, Daping Hospital Army Medical University, Chongqing, China; 7 Infinity Scope Biotechnology Co., Ltd., Hangzhou, China; 8 University College London Cancer Institute, University College London, London, UK; 9 College of Artificial Intelligence, Nanjing University of Aeronautics and Astronautics, Nanjing, China; 10 Chinese Institutes for Medical Research, Beijing, China; 11 James Thoracic Oncology Center, Ohio State University, Columbus, USA

**Keywords:** Lung adenocarcinoma lymph node metastasis, Genomic signatures, Tumor immune microenvironment, Spatial immunogenomic patterns

## Abstract

**Background:**

Lung adenocarcinoma (LUAD) with lymph node (LN) metastasis is linked to poor prognosis, yet the underlying mechanisms remain largely undefined. This study aimed to elucidate the immunogenomic landscape associated with LN metastasis in LUAD.

**Methods:**

We employed broad-panel next-generation sequencing (NGS) on a cohort of 257 surgically treated LUAD patients to delineate the molecular landscape of primary tumors and identify actionable driver-gene alterations. Additionally, we used multiplex immunohistochemistry (mIHC) on a propensity score-matched cohort, which enabled us to profile the immune microenvironment of primary tumors in detail while preserving cellular metaclusters, interactions, and neighborhood functional units. By integrating data from NGS and mIHC, we successfully identified spatial immunogenomic patterns and developed a predictive model for LN metastasis, which was subsequently validated independently.

**Results:**

Our analysis revealed distinct immunogenomic alteration patterns associated with LN metastasis stages. Specifically, we observed increased mutation frequencies in genes such as *PIK3CG* and *ATM* in LN metastatic primary tumors. Moreover, LN positive primary tumors exhibited a higher presence of macrophage and regulatory T cell metaclusters, along with their enriched neighborhood units (p < 0.05), compared to LN negative tumors. Furthermore, we developed a novel predictive model for LN metastasis likelihood, designed to inform non-surgical treatment strategies, optimize personalized therapy plans, and potentially improve outcomes for patients who are ineligible for surgery.

**Conclusions:**

This study offers a comprehensive analysis of the genetic and immune profiles in LUAD primary tumors with LN metastasis, identifying key immunogenomic patterns linked to metastatic progression. The predictive model derived from these insights marks a substantial advancement in personalized treatment, underscoring its potential to improve patient management.

**Supplementary Information:**

The online version contains supplementary material available at 10.1186/s40164-024-00574-8.

## Background

Lung adenocarcinoma (LUAD) metastasis, especially lymph node (LN) metastasis, plays a crucial role in influencing the clinical management and outcomes of patients [1–3]. However, understanding the complex molecular mechanisms and evolutionary dynamics underlying lung cancer metastasis remains challenging due to the substantial genomic and transcriptomic diversity both within and across patients [4]. Additionally, the limited number of large-scale genoproteomics studies in Eastern populations, specifically those focusing on primary tumor samples, hinders comprehensive characterization of metastatic processes. Studies relying on biopsy samples face inherent limitations in examining the driving events of metastasis, as they may not adequately capture the spatially distinct clonal or subclonal structures and molecular landscapes involved. Furthermore, unlike colorectal cancer [5] and breast cancer [6], which have been extensively studied in terms of metastatic trajectories, the patterns of metastasis in lung cancer remain relatively unexplored.

Broad-panel next-generation sequencing (NGS) has transformed oncology by enabling detailed insights into the molecular landscapes of tumors, identifying targetable driver-gene alterations, and informing prognosis [7, 8]. Despite these advancements, the genomic factors specifically associated with LN metastasis in East Asian patients with LUAD remain underexplored. Previous studies in Western cohorts have highlighted the role of genes, such as *STK11* and *SMACA4*, in LN metastasis [9]. To bridge this knowledge gap and further elucidate LN metastasis mechanisms in Chinese LUAD patients, we analyzed the genomic characteristics of primary tumors and their association with pathologic LN status in patients undergoing surgical treatment.

The tumor immune microenvironment (TIME) contributes significantly to the heterogeneity of LUAD and impacts tumor progression as well as LN metastasis. The spatial organization of immune cells within the TIME affects their functionality and the impact on the tumor ecosystem [10, 11]. Although single-cell analysis techniques like scRNA-seq and CyTOF have been employed to examine the cellular composition of tumors, they lack the required spatial resolution to comprehend the spatial distribution and functional relationships among different metaclusters within the TIME [12–14]. Multiplex immunohistochemistry (mIHC) provides a practical solution by enabling the simultaneous detection of multiple immune markers while preserving the spatial context, thus offering a spatially resolved single-cell phenotype [15, 16]. Cellular metaclusters, identified using mIHC, represent groups of cells that correspond to the primary cell types identified through our analysis. These metaclusters are formed by clustering similar cell types based on their phenotypic and molecular characteristics, allowing us to examine the complex cellular composition and interactions within the TIME. However, a comprehensive dimensional characterization of LN metastasis in LUAD through mIHC has yet to be explored.

The progression of LN metastasis in LUAD encompasses intricate spatial processes, encompassing tissue reorganization, invasion, and metastasis [17]. Previous investigation has highlighted the interplay between the genome and transcriptome in influencing LUAD evolution and metastasis [18]. Nevertheless, the exploration of metastasis progression requires a comprehensive understanding of spatial multi-omic patterns that are yet to be fully revealed. The TIME comprises a dynamic ecosystem consisting of tumor cells and their surrounding cellular components. Integrative spatial genomic and proteomic technologies present novel opportunities to explore cancer evolution at both molecular and spatial dimensions [19–21]. Utilizing NGS and mIHC, we identified spatial immunogenomic patterns that offer detailed characterizations of the TIME, cellular interactions, and micro-anatomical structures. Spatial genoproteomics exposed the distinctive relationship between TIME of primary tumor and pathological lymph node (pN) metastasis, providing insights into the underlying mechanisms that drive heterogeneity and opening new avenues for investigating LN metastasis in LUAD.

In this study, we conducted an integrative analysis of our cohort from Peking University People's Hospital (PKPH), consisting of patients with LUAD, by combining NGS with mIHC. Through the identification of spatial immunogenomic patterns, we performed a functional analysis of topological cellular neighbourhoods, which unveiled unique spatial interactions and associations with specific gene alterations to reveal the immunogenomic pattern of the dynamic LUAD ecosystem. Based on this, we developed a novel model with integrated immunogenomic characteristics to predict the clinical LN stage for patients with LUAD who are not eligible for surgery. These findings contribute to an improved understanding of the pathophysiological mechanisms involved in LN metastasis and emphasize the potential for personalized treatment strategies that target immunogenomic patterns in LUAD.

## Methods

### Patient cohort

This study received approval from the Institutional Review Board at Peking University People's Hospital (IRB NO.2021PHB182-001), and all patients provided written informed consent. The study, referred to as the PKPH NGS cohort, enrolled patients (n = 257) who underwent complete resection for pathologic stage I-III LUAD and NGS for cancer-related genes on their primary tumor between 2018 and 2020 using 363 (HR363, Berry Oncology, Beijing, China), HR457 (Berry Oncology, Beijing, China), or RS520 (Burning Rock Biotech, Guangzhou, China).

Preoperative radiology, including chest computed tomography (CT) scans and positron emission tomography (PET) scans if applicable, were performed to assess tumor size, pulmonary nodule morphology, lymph node involvement, and distant metastasis, which were used to evaluate clinical stages. Pathological stage analysis provided a more definitive assessment of the cancer's extent, as it is based on direct tissue analysis, including the evaluation of visceral pleural invasion and the gold-standard determination of the presence or absence of LN metastasis.

A cohort of 92 patients was selected for mIHC testing following propensity score matching within the broader NGS cohort (n = 257). Propensity score matching was performed using a 1:1 ratio to match LN metastasis cases with non-metastasis cases. The matching was conducted based on the nearest propensity score, using a caliper width of 0.2 standard deviations. The variables considered in the matching process included age, sex, comorbidities, smoking history, histology, and clinical stage.

### Tumor genomic analyses

We conducted target capture for the broad-panel NGS using commercial panels that included 363 (HR363, Berry Oncology, Beijing, China), 457 (HR457, Berry Oncology, Beijing, China), or 520 (RS520, Burning Rock Biotech, Guangzhou, China) cancer-related genes, covering 1.21 megabases (Mb) of the human genome. The protocols for genomic DNA extraction, targeted/whole-exome sequencing library preparation, and variant calling were described in previous publications [22].

We assessed the genomic alteration count in the broad-panel NGS cohort, defined as the sum of nonsynonymous somatic mutations, copy number variations (CNVs), and gene fusions. Additionally, we calculated the mutant-allele tumor heterogeneity (MATH) score based on the dispersion of variant allele frequency (VAF) distribution. A higher MATH score indicated a tumor with higher intratumor heterogeneity (ITH).

We applied the MutSigCV and dNdScv algorithm to infer significantly mutated genes (q < 0.1 in any caller and nonsilent mutations n ≥ 5) in the broad-panel NGS cohort (n = 257), including 53 pN positive and 204 pN negative cases. For oncogenic pathway analyses, we retained only functional alterations labeled as oncogenic, likely oncogenic, or predicted oncogenic in the OncoKB database, discarding variants of unknown significance. Therapeutic actionability information was also annotated using the OncoKB database, and each genomic alteration was stratified into one of four levels according to its clinical implication.

### mIHC section preparation

The mIHC staining was performed by Alphaxbio Biotechnology Co., Ltd. (Beijing, China), using a panel of six markers: PANCK, CD4, CD8, FOXP3, CD68, and PD-L1.

The 4-μm thick FFPE LUAD tissue slides were subjected to deparaffinization in xylene for 30 min followed by rehydration in absolute ethyl alcohol for 5 min (twice), 95% ethyl alcohol for 5 min, and 75% ethyl alcohol for 2 min sequentially. The slides were washed three times with distilled water. Heat-induced epitope retrieval was performed using a microwave oven, and the slides were immersed in boiling EDTA buffer (PH9.0; ZLI-9069; Zsbio, Beijing, China) for 15 min. Antibody Diluent/Block (72424205; Akoya Biosciences, DE, USA) was used for blocking before the detection of each panel, which included a total of 6 markers: PANCK, CD4, CD8, FOXP3, CD68, and PD-L1. The mIHC staining was carried out at Alphaxbio Biotechnology Co., Ltd. (Beijing, China).

Primary antibodies including FOXP3 (ab20034; Abcam, Cambridge, UK), PANCK (ab7753; Abcam, Cambridge, UK), CD8 (ab237709; Abcam, Cambridge, UK), CD68 (ab192847; Abcam, Cambridge, UK), CD4 (ab133616; Abcam, Cambridge, UK), and PDL1 (ab237726; Abcam, Cambridge, UK) were incubated at 37 °C for 1 h. The slides were then incubated with Opal Polymer HRP Ms + Rb (2414515, Akoya Biosciences, USA) for 10 min at 37 °C, followed by visualization using the Opal Seven-Color IHC Kit (NEL797B001KT, Akoya Biosciences, USA), with the correspondence between primary antibodies and fluorophores as XTSA520, XTSA540, XTSA570, XTSA620, XTSA650, and XTSA690. After each cycle of staining, heat-induced epitope retrieval was performed to remove all antibodies, including primary antibodies and Opal Polymer HRP Ms + Rb. Finally, the slides were counterstained with DAPI for 5 min and enclosed in Antifade Mounting Medium (I0052; NobleRyder, Beijing, China).

### mIHC analysis and cellular metaclusters identification

To analyze the tissue sections, mIHC images with high resolution in the image dataset were randomly selected as training samples to build an algorithm of tissue segmentation, cell segmentation, phenotyping tool, and positivity score using inForm Advanced Image Analysis software (inForm 2.5.0; Akoya Biosciences, USA). The algorithm was then applied in batch analysis of all the images. These procedures generated a single-cell dataset. PANCK, CD4, CD8, FOXP3, CD68, and PD-L1 were used for the subclass division of tumor cells and immune cells. Based on the extensive high-throughput dataset, we have applied harmony to eliminate the batch effect prior to conducting downstream analysis, a method that demonstrated superior performance compared to combat in this scenario. The harmony algorithm performs batch correction by iteratively clustering and correcting the positions of cells in Principal Component Analysis (PCA) space. PCA was initially performed on the high-dimensional matrix. The top 20 principal components were then input into the HarmonyMatrix function within the Harmony package to eliminate the batch effect. In order to reduce the dimensionality of the data for visualization, Uniform manifold approximation, and projection (UMAP) was employed. Additionally, classical markers were utilized for the manual annotation of cell metaclusters.

### The TIME subtypes analysis with cellular metaclusters

To identify TIME subtypes, we applied the consensus non-negative matrix factorization (CNMF) algorithm [23], an unsupervised clustering method chosen for its balance between classification stability and similarity optimization. The analysis was performed using R 4.0.2 (R Core Team, Vienna, Austria), with input data derived from cellular metacluster identification profiles. The CNMF algorithm was optimized for both stable classification and similarity within the data. The number of subtypes was defined as two, and the algorithm was run for 50 iterations. These parameters were selected to ensure robust clustering while optimizing for both stability and inter-cluster similarity. For samples that were assigned to the subtypes, the final category label was determined by integrating the group information from all involved cellular metacluster by ROI (n = 597).

The CNMF method generates group assignments, which were output as cluster labels and used to create visualization, including a consensus heatmap and silhouette score plots. The consensus heatmap, based on the distance matrix, was used to assess clustering coherence, with rows and columns organized according to clustering results. The silhouette plot, which evaluates cluster quality, showed that higher silhouette widths indicated better cluster definition, further validating the balance between classification stability and similarity in our analysis. By employing this method, we were able to identify and characterize key TIME subtypes, specifically those associated with LN metastasis in lung adenocarcinoma primary tumors.

### Cellular neighbourhood (CN) analysis

We conducted Delaunay triangulation-based graph construction utilizing the Euclidean distance between their X/Y coordinates, followed by 20-nearest neighbourhood aggregation, and subsequently clustered cells to ascertain the CN of each cell [24]. These windows were then clustered based on their compositions regarding all cell metaclusters, using K-means clustering. Setting k = 10, each cell’s CN was subsequently assigned according to the CN of its surrounding window [12, 25]. To validate the CN assignment, we overlaid the Voronoi allocation graphs onto the original tissue mIHC images.

### Univariable and multivariable logistic regression analysis

Initially, we conducted univariate analyses regressing the LN status (pN positive or negative) on the clinicopathologic features and genomic alterations. The formula for univariable logistic regression was expressed as:

$$logit(pr)={\beta }_{0}+{\beta }_{1}X$$

where: *pr* is the probability of the LN metastasis occurring, *β*_*0*_ is the intercept term, and *β*_*1*_ is the coefficient for the predictor clinicopathologic or genomic variable.

The top clinicopathologic or genomic factors associated with LN metastasis in univariate analysis were included as covariates in multivariate logistic regression. The formula for multivariable logistic regression was expressed as:

$$logit(pr)=-3.07+(-1.28\times Nodule\_type)+(-0.035\times Tumor\_size)+(0.69\times Histologic \,subtype)+(-0.46\times Path\_STAS)+(0.72\times Path\_VPI)+(1.35\times Path\_LVI)+(0.084\times EGFR)+(-0.34\times TP53)+(-0.83\times RBM10)+(0.17\times STK11)+(0.75\times ATM)+(1.33\times HGF)+(0.97\times KEAP1)+(2.66\times PIK3CG)$$

### The imgene model construction

To predict the LN stage, we implemented each classifier using the scikit-learn (1.0.1, https://scikit-learn.org/stable/) library in Python. The coefficients were calculated as shown in Fig. 6B. We selected the top 10 coefficients from both the mIHC image features (ImFeatures) and the genomic features (GeneFeatures) to construct the models. After removing samples with unmatched ImFeatures, we obtained a dataset consisting of 83 patients with mIHC and NGS data. Among the classifiers, ImGene Support Vector Machines (SVM) demonstrated the highest performance with an AUC of 0.86, F1 Score of 0.83, and Accuracy of 0.82, surpassing Random Forest, ImFeatures SVM, GeneFeatures SVM, and Gradient Boosting.

where, $$Z = 3.51 + \left( {\beta _{{Im}} \times ImFeatures} \right) + \left( {\beta _{{Gene}} \times GeneFeatures} \right)$$

${\beta }_{Im}$ Is the coefficient for the ImFeatures, ${\beta }_{Gene}$ is the coefficient for the Genefeatures (Fig. 6C).

To construct individual models based on IHC image and genomic features, SVM was implemented using three parameters: (1) C, which represents the penalty coefficient, (2) the Kernel function, and (3) Gamma. The best combination of these parameters was determined using Leave-One-Out Cross-Validation with the training dataset. The cutoff value for classification was set at the point with the highest accuracy in the validation set. In order to obtain the optimal model, logistic regression models were constructed using the predictive scores from the individual models as input features. This integration of the performance of each model was based on both the discovery and validation datasets. The probability of the LN status in a patient was calculated as follows:

$$Pr(ImGene)=exp(Z)/(1+exp(Z))$$

### Validation dataset

An independent external dataset comprising 61 patients treated with complete resection approved by ethics number IRB NO.2021PHB182-001 was collated as the Peking University People’s Hospital Thoracic Oncology Institution (PKTOI) cohort. All patients provided informed consent for sample collection and all participants consented to the publication of research results. These cases were selected due to the availability of NGS and mIHC data. Clinical details for these 61 cases are summarized in Supplementary Table 4.

### Statistical analyses

Clinicopathologic features were summarized as either medians with interquartile ranges (IQRs) or frequencies with percentages. To compare these features across the two LN groups, we used the Mann–Whitney U test for continuous variables, while categorical variables were assessed using the Fisher’s exact test. For genomic variables, the Fisher’s exact test with False Discovery Rate (FDR) adjustment was applied to account for multiple comparisons. To identify independent predictors of LN metastasis, we conducted both univariate and multivariate negative binomial regression analyses, incorporating clinicopathologic and tumor genomic characteristics.

Data obtained from mIHC were analyzed using GraphPad software, with statistical tests selected based on data distribution and variability. The results are presented as means ± standard error of the mean (SEMs). The Spearman rank correlation test was used to explore relationships between spatial and quantitative variables. The Mann–Whitney U test and Kruskal–Wallis H test were used to compare continuous variables between two and more groups respectively. Categorical data were analyzed using the Chi-squared test or Fisher’s exact test.

All statistical analyses were performed using R 4.0.2 (R Core Team, Vienna, Austria). The p < 0.05 (or FDR q < 0.1) was considered statistically significant.

## Results

### Genomic features associated with pathologic ln metastasis in the PKPH Cohort

To identify genomic features associated with pathologic LN metastasis in LUAD primary tumors, we conducted the broad panel NGS analysis in the PKPH cohort. The cohort included a total of 257 patients who met the inclusion criteria (Table 1), with 54.8% (n = 141) were female and 71.6% (n = 184) were never smokers. Diagnostic CT scans revealed a solid morphologic appearance in the primary tumors of 53% (108/204) of pN-negative cases and 90% (48/53) of pN-positive cases, showing a significant difference (Fisher’s exact test, p < 0.001). The total primary tumor size, as measured on CT scans, was significantly larger in pN positive tumors (median [IQR], 2.9 [2.3–3.6] cm) compared to pN negative tumors (2.3 [1.6–3.1] cm, Mann–Whitney U, p < 0.001). The majority of patients (n = 193 [75%]) had clinical stage I disease, while 22% (n = 10) had clinical stage II disease. Pathologic review revealed that 79% (n = 204) of resected tumors were pN negative, while 21% (n = 53) were pN positive. The acinar/papillary predominant histologic subtype was significantly lower in pN positive tumors (59%) compared to pN negative tumors (23%, Fisher’s exact test, p < 0.001). Lymphovascular invasion and visceral pleural invasion were significantly higher in pN positive tumors (38/53 vs. 52/204, 30/53 vs. 35/204 for pN positive vs. pN negative, respectively, Fisher’s exact test, p < 0.001). The clinicopathologic features of the patients are summarized in Table 1.

**Table 1:** Clinicopathologic characteristics of the PKPH NGS cohort

Characteristics	pN negative	pN positive	p value
n	204	53	0.548
Age, median (IQR)	61 (54, 68)	63 (56, 68)	
Sex, n (%)			
Male	93 (45.6%)	23 (43.4%)	0.775
Female	111 (54.4%)	30 (56.6%)	
Smoking, n (%)			
No	146 (71.6%)	38 (71.7%)	0.985
Yes	58 (28.4%)	15 (28.3%)	
Nodule_type, n (%)			
Solid	108 (52.9%)	48 (90.6%)	** < 0.001**
Subsolid	96 (47.1%)	5 (9.4%)	
Histologic subtype, n (%)			
ACI/PAP	152 (74.5%)	23 (43.4%)	** < 0.001**
MIP/SOL	30 (14.7%)	29 (54.7%)	
LEP	7 (3.4%)	0 (0%)	
Others	15 (7.4%)	1 (1.9%)	
Path_STAS, n (%)			
No	181 (88.7%)	44 (83%)	0.262
Yes	23 (11.3%)	9 (17%)	
Path_VPI, n (%)			
No	169 (82.8%)	23 (43.4%)	** < 0.001**
Yes	35 (17.2%)	30 (56.6%)	
Path_LVI, n (%)			
No	152 (74.5%)	15 (28.3%)	** < 0.001**
Yes	52 (25.5%)	38 (71.7%)	
Stage, n (%)			
IB	49 (24%)	0 (0%)	** < 0.001**
IIIA	2 (1%)	32 (60.4%)	
IA2	79 (38.7%)	0 (0%)	
IIB	4 (2%)	17 (32.1%)	
IA1	33 (16.2%)	0 (0%)	
IA3	32 (15.7%)	0 (0%)	
IIA	5 (2.5%)	0 (0%)	
IIIB	0 (0%)	4 (7.5%)	

Bold indicates statistically significant results (*p* < 0.05)

Data are number (%) or median (interquartile range)，bold indicates statistically significant results (p < 0.05).

*PKPH* Peking University People’s Hospital, *NGS* next-generation sequencing, *ACI* acinar, *LEP* lepidic, *LVI* lymphovascular invasion, *PAP* papillary, *MIP* micropapillary, *SOL* solid, *STAS* spread through air spaces, *VPI* visceral pleural invasion

**Fig. 1:**
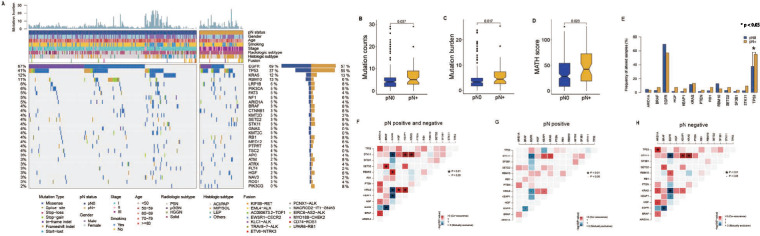
Genomic Features Associated with Pathologic LN Metastasis. **A** OncoPrint displayed the gene alterations across the entire cohort, stratified by pathologic lymph node (LN) status. **B** Mutation frequencies in key driver genes were compared based on pathologic LN status. **C**-**E** Box plots showed the distribution of tumor mutation counts, TMB, MATH scores according to pathologic LN status. *LN* lymph node, *TMB* tumor mutational burden (mutations per megabase), *MATH* mutant-allele tumor heterogeneity

In the PKPH NGS cohort, the most commonly detected drivers were *EGFR* (66%), *TP53* (41%), and *KRAS* (12%). We identified 13 driver genes with a FDR of 0.1. Notably, *TP53* mutations were significantly more prevalent in pN positive primary tumors compared to pN primary negative tumors (55% vs. 37%, Fisher’s exact test, q < 0.001, Fig. 1B). Furthermore, *PIK3CG* alterations were significantly more common in pN positive primary tumors compared to pN negative ones (7.5% vs. 0.5%, Fisher’s exact test, p < 0.05, Supplementary Fig. 1). Conversely, no statistically significant differences were observed in the prevalence of mutations between pN positive and negative primary tumors for other genes analyzed, including *EGFR, KRAS, RBM10, RB1, STK11, SETD2, SF3B1, PTEN, HGF, BRAF, ARID1A, and KEAP1*.

We performed subsequent analysis to examine genomic differences in the PKPH NGS cohort of LN metastasis, focusing on the tumor mutation count, tumor mutational burden (TMB), and mutant-allele tumor heterogeneity (MATH) score (Fig. 1C-E). The results demonstrated a significant disparity in the tumor mutation counts between pN positive and pN negative tumors (median [IQR], 6.0 [3.0-9.0] vs. 4.0 [2.0-6.0], p = 0.037, Fig. 1C). The TMB was notably higher in pN positive tumors (median [IQR], 4.74 [2.63–7.38] mutations/Mb) compared to pN negative tumors (median [IQR], 3.16 [1.75–4.92] mutations/Mb, p = 0.017, Fig. 1D). The MATH score exhibited a significantly higher value in pN positive tumors (median [IQR], 53.49 [19.03–72.51]) compared to pN negative tumors (median [IQR], 45.67 [8.81–54.45], p = 0.02, Fig. 1E), suggesting a relatively elevated level of intratumor heterogeneity in pN positive tumors.

The present study investigated the co-occurrence and mutual exclusivity patterns of *EGFR* and several key genes in a large panel of NGS data. Our analysis revealed that *EGFR-RBM10, KRAS-STK11, KEAP1-STK11, KEAP1-SETD2, KEAP1-KRAS, HGF-STK11, HGF-KRAS, BRAF-SETD2, ARID1A-TP53, ARID1A-PTEN, ARID1A-KRAS, ARID1A-HGF, ARID1A-BRAF, and BRAF-SETD2* showed significant co-occurrence, whereas *EGFR-KEAP1*, *EGFR-STK11, EGFR-KRAS,* and *BRAF-EGFR* exhibited mutual exclusivity across the entire cohort (p < 0.05, Supplementary Fig. 2A, Supplementary Table 2.1). In the subgroup of pN positive tumors, we observed co-occurrence for *KRAS-STK11, KEAP1-STK11,* and *KEAP1-KRAS*, whereas *EGFR-STK11, BRAF-EGFR,* and *EGFR-KRAS* were mutually exclusive (p < 0.05, Supplementary Fig. 2B, Supplementary Table 2.2). Furthermore, we identified *EGFR-RBM10, KRAS-STK11, KEAP1-STK11, KEAP1-KRAS, ARID1A-TP53, ARID1A-KRAS, ARID1A-HGF, ARID1A-BRAF,* and *BRAF-SETD2* as significant mutual co-occurrence drivers in pN negative tumors, while *EGFR-STK11, EGFR-KRAS,* and *BRAF-EGFR* exhibited mutual exclusivity in pN negative tumors (p < 0.05, Supplementary Fig. 2C, Supplementary Table 2.3).

Our analysis, which integrated alteration frequency, tumor mutation count, TMB, and MATH score, reinforced the association between genomic alterations and LN metastasis in LUAD.

### Preoperative clinicopathologic and genomic features associated with pathologic LN Metastasis

To identify clinicopathologic and genomic features associated with the presence of pathologic LN metastasis, we compared pN positive primary tumors with pN negative primary tumors by logistic regression analyses. Univariable logistic regression revealed significant associations between several clinicopathologic and genomic factors and LN metastasis in the NGS cohort (Supplementary Table 1, Supplementary Fig. 1A). Specifically, we observed significant differences (p < 0.001) in primary tumor appearance and size on CT, lymphovascular invasion, and visceral pleural invasion. Additionally, mutations in *TP53* (p = 0.028) and *PIK3CG* (p = 0.013) were notably and significantly correlated with LN metastasis.

The further results of the multivariable regression analysis performed by above features, showed that the tumor morphologic appearance on CT (odds ratio [OR], 0.276, 95% confidence interval [CI], 0.085–0.89, p = 0.032), tumor size on CT (OR, 1.036, 95% CI, 1.001–1.071, p = 0.042), LVI (OR, 3.89, 95% CI, 1.63–9.29, p = 0.02), and the presence of *PIK3CG* mutation compared to wild-type (p = 0.049) were associated with pathologic LN metastasis (Supplementary Table 1, Supplementary Fig. 1B).

This study reinforced our understanding of genomic variables, such as *PIK3CG*, linked to LN metastasis in LUAD. Further research is necessary to fully elucidate their role in pathogenesis.

### Oncogenic pathways and therapeutic actionabilities associated with pathologic LN Metastasis

**Fig. 2:**
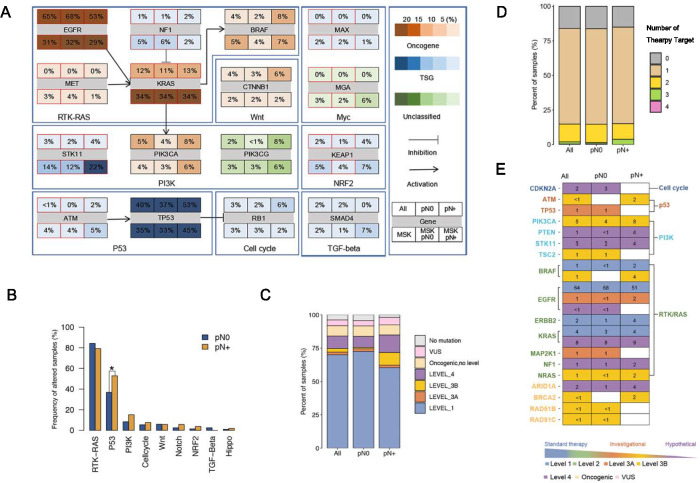
Oncogenic Pathway and Therapeutic Actionability Associated with Pathologic LN Metastasis. **A** Somatic mutation comparisons were made between PKPH cohort and the MSK cohorts. Each gene box represented the mutational frequencies of genes in the entire cohort, pN0 (negative) and pN + (positive) groups, and the MSK cohort as a whole, including pN0 and pN + groups, as shown in the graph. The color scale bar indicated mutation frequency from 0 to 100%. Genes were grouped by signaling pathways related to genome maintenance mechanisms. Interactions between genes were indicated by arrows. **B** Comparison of alteration frequency of oncogenic pathways among pathologic LN status. Pathways with significant differences from Fisher’s exact test were labeled with asterisks (FDR q <0.1), while TP53 significant difference is highlighted (p < 0.05). **C** Frequency of samples with actionable alteration by pathologic LN status. **D** Level of evidence versus pathologic LN status. Samples were classified by the alteration that carried the highest level of evidence. **E** Frequency of the number of actionable alterations versus pathologic LN status. *TSG* tumor suppressor gene, *FDR* false discovery rate, *VUS *variant of uncertain significance

To further examine the association between oncogenic pathways and LN metastasis in the analysis of genomic variables above, we conducted a comparison of oncogenic signaling pathway alteration frequencies between pN positive and negative samples in the PKPH NGS cohort. Results of the Fisher’s exact test revealed a significant increasing trend in the frequency of p53 pathway alterations from pN negative to pN positive samples (p = 0.04, Fig. 2B). Nevertheless, no significant difference was found probably due to limited sample size. The co-occurrence and mutual exclusivity patterns analysis of oncogenic pathways is presented in Supplementary Fig. 2.

A further comparison of somatic mutations in genes related to genomic integrity between our PKPH NGS cohort and the Western MSK cohort (n = 429) was conducted to identify any significant differences or similarities in mutation profiles across different populations. *KRAS* (12%), *NF1* (1%), and *BRAF* (4%) of the receptor tyrosine kinase (RTK)/RAS pathway, *STK11* (3%) of the phosphatidylinositol 3-kinase (PI3K) pathway, and *KEAP1* (2%) of the NRF2 pathway had lower mutation frequencies, while *EGFR* (65%) of the RTK/RAS pathway, *PIK3A* (5%) of the PI3K pathway, and *TP53*(40%) of the p53 pathway had much higher mutation frequencies in our cohort than in Western populations (Fig. 2A). *PIK3A* (8%) of the PI3K pathway was detected with higher mutation frequencies in the pN positive group compared with those in the negative group (4%) in our PKPH cohort and MSK cohort (6% vs 3%). The same observations were also identified in *BRAF* of the RTK/RAS pathway in the PKPH cohort (8% vs 2%) and MSK cohort (7% vs 4%). These findings suggested potential differences in mutation patterns and highlighted the importance of RTK/RAS and PI3K pathway-related genetic mechanisms in primary tumors, particularly in relation to LUAD LN metastasis, between our PKPH cohort and the MSK cohort.

A total of 260 actionable alterations were identified using the OncoKB database, spanning 18 genes and consisting of 184 (71%) level 1, 7 (3%) level 3A, 23 (9%) level 3B, and 46 (17%) level 4 alterations (Fig. 2C-E). The RTK/RAS pathway had the highest number of actionable alterations (84% [218/260]), of which 84% (184/218) had level 1 evidence. At the sample level, actionable alterations were identified in 84% (216/257) of the 257 samples, of which 83% (180/216) had level 1 evidence. Notably, pN positive tumors showed a higher frequency tendency of level 1 actionable targets (73% vs. 60% for pN + vs. pN0, p = 0.094). The mean number of actionable alterations per sample among subgroups did not differ significantly (1.038 vs. 1.005, all p > 0.05).

Our analyses of oncogenic pathways and therapeutic targets corroborated existing data, underscoring molecular mechanisms linked to LN metastasis in LUAD. These findings provided foundational insights into diverse genomic mutation patterns.

### Identification of cellular metaclusters related to ln metastasis in TIME

**Fig. 3:**
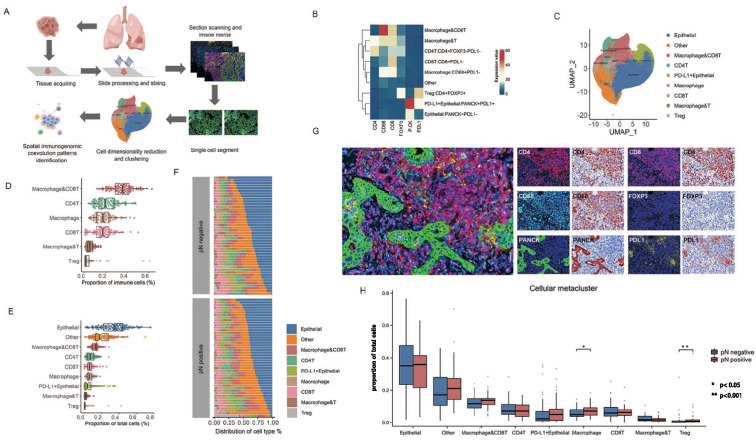
mIHC Defined the Spatial Landscape of LN Metastasis. **A** A schematic depicted the process of mIHC image acquisition from 92 patients with LUAD, including tissue preparation, antibody staining, image acquisition and assembly, single-cell segmentation, cell dimensionality reduction, clustering, and LN stage analysis. **B** Heatmaps showed the z-scored mean marker expression of 9 metaclusters, with their frequency distribution patterns across different patients displayed in the box plot on the right. Pixel intensities were transformed using an asinh transformation with a cofactor of 1. **C** A UMAP plot represented the 9 annotated metaclusters. **D**, **E** The prevalence of 9 cell types across 92 patients with LUAD was shown as a proportion of immune cells (**D**) and total cells (**E**). **F** A waterfall plot depicted the distribution of 9 cell types across LN status.** G** Representative mIHC images from LN metastasis primary tumor samples were analyzed using the 6-marker panel. **H** The prevalence of 9 metaclusters was compared across LN stages. The comparison of macrophage and Treg cells between pN positive and negative tumors was shown: *p < 0.05, **p < 0.001. Data were presented as means ± SEMs. Statistical analysis was conducted using the Mann–Whitney U test

To explore the spatial cellular composition in primary tumors associated with LN metastasis, we utilized mIHC to analyze primary tumor samples from 92 patients after propensity score matching in PKPH NGS cohort. We optimized a 6-antibody panel to identify cancer cells, stromal cells, as well as innate and adaptive immune lineages with diverse functional characteristics (Fig. 3A).

We utilized a dimensionality reduction approach to classify 9 distinct cellular metaclusters (Fig. 3B, C), and the statistical significance of differences between these metaclusters was evaluated using the Mann–Whitney U test. pN positive tumors exhibited a higher percentage of immune infiltrate (36.5%) compared to pN negative tumors (31.1%, Fig. 3D, E). Notably, the disparity was primarily driven by alterations in the macrophage (7.1% vs. 4.8%, p < 0.05) and Treg (0.85% vs. 0.29%, p < 0.001) populations (Fig. 3G), while no significant variations in the overall frequency of total cells were observed between different LN metastasis statuses. No significant differences were observed in the average frequency of CD4^+^T, CD8^+^T, and epithelial cellular metaclusters in total cells between pN positive and negative tumors. Our analyses involved the classification of 9 distinct cellular metaclusters in primary tumor samples of LUAD, emphasizing the notable contributions of macrophages and Tregs proportions in the TIME [26].

**Fig. 4:**
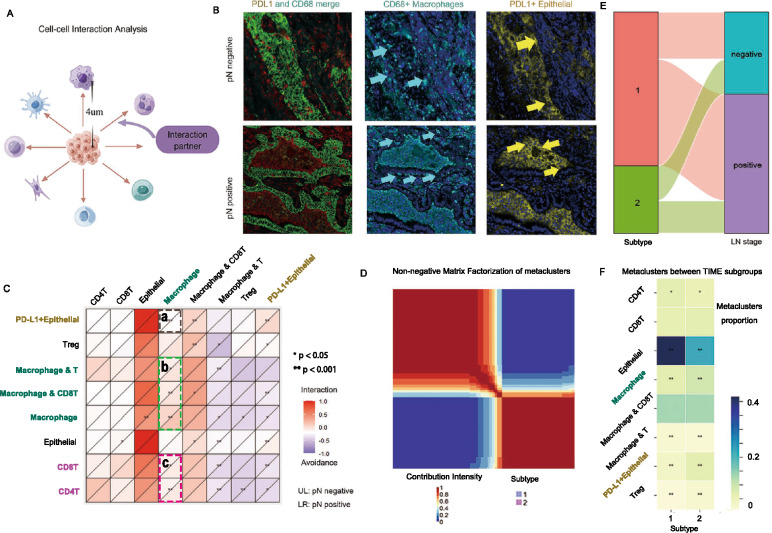
Variability in mIHC Distributions Across Clinical Variables and Cellular Interaction Profiles Across the LN Stage in LUAD. **A** An interaction partner detection model was used to identify neighboring cells within 4 µm of central cells. **B** Segmented images showed a decrease in interactions between PD-L1 positive epithelial cells and macrophages in pN positive LUAD. **C** A heatmap depicted significant pairwise cellular interactions (red) or avoidance (blue) across LN stage subgroups. The boxes highlighted associations referenced in the text. **D** Two distinct subtypes were identified within the tumor immune microenvironment (TIME) of LN metastasis primary tumors through unsupervised analysis with Non-negative Matrix Factorization: an epithelial type and a macrophage and immune-regulatory cell-enriched type. **E** An association was found between the frequency of LN metastasis and the distribution of the two TIME subtypes (p < 0.001). **F** The prevalence of cellular metaclusters was compared between TIME subtypes: *p < 0.05, **p < 0.001. Data were presented as means ± SEMs. Statistical analysis was conducted using the Mann–Whitney U test

### Spatial cellular interactions related to ln metastasis and cellular metaclusters analyses reveal two time subtypes

In order to understand the cellular architecture and spatial organization beyond the metaclusters frequency of LN metastasis in LUAD, we analyzed the communication patterns between individual metaclusters by quantifying the spatial relationships (Fig. 4A). The distribution of the numbers of cellular connections and pairs of cell distance (μm) was presented in Supplementary Fig. 3. Furthermore, we determined the probability of interaction or avoidance behaviors between pairs of cells across tumor architectures in both pN negative and pN positive tumors (Fig. 4B). By comparing the cellular interactions within metaclusters between pN negative and pN positive tumors, macrophages were indicated at the center exhibited significant differences in spatial cell interactions with a broader range of other subclusters, including PD-L1 positive epithelial cells (Fig. 4C, box a), macrophages and T, macrophages and CD8^+^T (Fig. 4C, box b), CD4^+^T and CD8^+^T (Fig. 4C, box c). The interactions between macrophages and tumor epithelial cells in the primary tumor lesion of pN positive tumors were higher, which aligns with previous research highlighting the role of macrophages in the TIME [27, 28]. These analyses painted an overall picture of how cellular metaclusters interactions shift among LN metastasis in LUAD and exemplified how spatial relationships, rather than cell frequency alone, are important to understand patterns of spatial TIME biology.

To investigate the patterns involved in cellular metaclusters and genetic characteristics, we identified two distinct subtypes within the TIME of primary tumors with LN metastasis using non-negative matrix factorization algorithm (Fig. 4D). These subtypes consisted of an epithelial subtype (subtype 1) and a macrophage and Treg enriched subtype (subtype 2), as shown in Figure F. We found a significant association between the frequency of LN metastasis and the distribution of these two subtypes (Fisher’s exact test, p < 0.001, Fig. 4E), with subtype 2 demonstrating a higher propensity for advanced LN stages. This novel approach [29], which utilizes nine metaclusters for classification, provides a new perspective on the heterogeneity of the LUAD TIME in relation to LN metastasis.

Using the Mann–Whitney U test to analyze differences in metaclusters between subtypes, we found significant differences in the proportions of CD4^+^T cells between subtype 1 (mean = 0.0649) and subtype 2 (mean = 0.0539) with p = 0.0166, indicating distinct roles for CD4^+^T cells across these subtypes. In contrast, the proportions of CD8^+^T cells did not differ significantly between the subtypes (subtype 1 mean = 0.0600, subtype 2 mean = 0.0651, p = 0.3235, Fig. 4F).

The observed difference in epithelial cells was particularly significant, with subgroup 1 showing an average value of 0.4171, compared to 0.2195 in subgroup 2 (p < 0.001), highlighting the critical role of epithelial cells in the LN metastatic TIME. Similarly, macrophages showed significant distribution differences (subtype 1 mean = 0.0662, subtype 2 mean = 0.0919, p < 0.001, Fig. 4F), suggesting divergent functions across two TIME subtypes.

Further insights were gained from the analysis of gene mutation frequencies across the two distinctive TIME subtypes (Supplementary Fig. 4C). The *ATM* and *PIK3CG* genes exhibited the lower mutation frequency in subtype 1 and higher in subtype 2 (*ATM* p = 0.013, *PIK3CG* p = 0.016), potentially linked to the cell’s DNA damage repair capacity and activation of immune inflammatory cells. Both *BRD4* and *KMT2B* genes showed higher mutation frequencies in subtype 2 (*BRD4* p = 0.041, *KMT2B* p = 0.041), indicating that these epigenetic regulators might play different roles across LUAD TIME subtypes. The *CTNNB1* gene showed a higher mutation frequency in subtype 1 (p = 0.046), associated with potential changes in the Wnt signaling pathway, impacting cell fate and proliferation.

In the subtype 1, we observed co-occurrence for *EGFR-TP53, KRAS-TP53* and *EGFR-CTNNB1* (p < 0.05, Supplementary Fig. 4B). Furthermore, we identified *KRAS-TP53* and *EGFR-PIK3CG* as significant mutual co-occurrence drivers in subtype 2 (p < 0.05, Supplementary Fig. 4B).

Our analyses offered early insights into the dynamic interactions of cellular metaclusters in LUAD primary tumors, focusing on LN metastasis. The findings suggested that spatial relationships, rather than cell frequency alone, were essential for understanding spatial TIME biology [30, 31].

### Analysis of distinct CN alterations between ln negative and positive LUAD patients

**Fig. 5:**
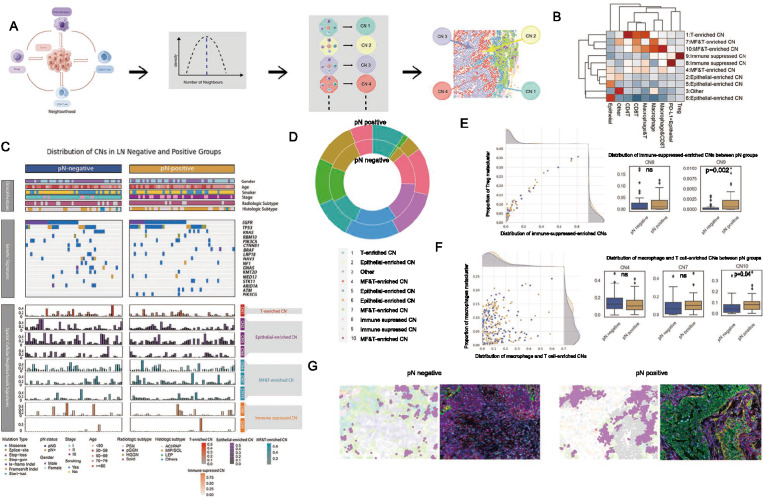
Correlations of LUAD TIME Cellular Function Units with Patient LN Status. **A** The analysis schedule for cellular neighbourhoods (CNs) was outlined. CNs were defined by the center cell, primary neighbours, and secondary neighbours. The peak number of cells within a CN was approximately 10. **B** Ten major types of CNs were first annotated based on the major cell type within each CN and were clustered according to the scaled frequency of each cell type within the CNs. **C** An overview of clinical, genetic alterations, and CN signatures was provided for 92 patients. **D** The inner and outer layers of the donut chart respectively represented the differences in CN distribution between pN negative and positive cases. **E** Statistical analysis showed a correlation between immune-suppressed-enriched CNs (CN 8 and 9) and the Treg component, highlighting differences between pN negative and positive cases: CN 8 (p > 0.05) and CN 9 (p = 0.002). **F** Statistical analysis showed a correlation between macrophage and T cell-enriched CNs (CN 4, 7, and 10) and the macrophage component, with differences observed between pN negative and positive cases: CN 4 and 7 (p > 0.05), CN 10 (p = 0.04). **G** In the mIHC image, a Voronoi plot with distinct colors represented the distribution of CN 9 (gray) and CN 10 (purple), both of which were more abundant in pN positive cases. *CN* cellular neighbourhood

In the preceding section, we conducted an analysis of the variations in cellular metacluster frequencies observed between pN negative and positive primary tumors in patients with LUAD. To determine specific cellular interacting critical for the LN metastasis, we conducted CN and cell interaction assessments. We annotated the CNs based on the major cellular cluster within the TIME (Fig. 5A, B, C). First, a cellular neighbourhood was designated as the ten closest neighbours surrounding the central cell. The cellular neighbourhoods were defined to group cells, encompassing the central cell along with its immediate neighbours within a 4 µm radius, as well as the secondary neighbours of these primary neighbours. Such definition is more suitable for the CN with > 10 neighbours and able to remove artificial neighbours when separate cells were in distant location in Fig. 5A and 5B. The distribution of the CN is presented in Supplementary Fig. 5A. Recent CN analysis framework established by Schürch et al. [32] and Sheng et al. [24] were adapted.

To determine various functional cellular neighbourhood units, these neighbourhoods were annotated based on the primary cellular clusters (Fig. 5B, D). Our analysis revealed distinct CN functional units, including epithelial-enriched CNs (Epithelial-CN, 2, 5, 6), macrophage and T cell-enriched CNs (MF and T-CN, 4, 7, 10), immune-suppressed-enriched CNs (Immune-suppressed-CN, 8, 9), and CNs enriched in CD8^+^ and CD4^+^ T cells (T-CN, 1). Notably, the abundance of Immune-suppressed-CNs exhibited coherent links with the Treg metacluster proportion (Fig. 5E), whereas macrophage and T cell-enriched CNs showed links with the macrophages metacluster proportion (Fig. 5F).

We used the Mann–Whitney U test to compare CNs between pN-positive and pN-negative groups and observed a significant increase in the number of CN9 exclusively in the pN positive LUAD patients primary tumor (Fig. 5E). CN10 also increased in pN positive group of LUAD patients primary tumor (p = 0.04, Fig. 5F). However, However, the CN4 and CN7 showed no LN metastasis deference (p > 0.05, Fig. 5F). In contrast, no such trend was observed in the epithelial-enriched CNs (Epithelial-CN, 2, 5, 6) and T-CN (p > 0.05, Supplementary Fig. 5B, C). These results suggested that LUAD topological TIME units could be novel biomarkers for LN metastasis. In addition, macrophage and Tregs organized CNs that play a critical role in the TIME of LN positive primary tumor.

We applied a color-coding scheme to each mIHC image based on the CN composition, and utilized a Voronoi plot to generate a topology map for each mIHC image (Fig. 5G), employing the methodology from our previous research. The resulting topology map, encoded with CN information, accurately represented the mIHC image.

Subsequently, our analysis uncovered significant associations between the abundance of CNs and gene mutations (Supplementary Fig. 5D). Specifically, *PIK3CG* mutations consistently showed a higher enrichment of macrophage and T cell-enriched CN (CN10) and T-CN (CN1), as well as a lower enrichment of epithelial-CN (CN5, CN6). Conversely, *KRAS* mutations were associated with a lower enrichment of MF and T-CN (CN10) and T-CN (CN1), while exhibiting a higher enrichment of epithelial-CN (CN5).

Our spatial proteomic analysis revealed novel findings, including macrophage functional heterogeneity and T cell enrichment associated with *PIK3CG* mutation in LUAD primary tumors. By integrating genetic mutation profiles with proteomic data, we built a comprehensive spatial genoproteomics landscape of LN metastatic LUAD primary tumors.

### Predicting ln metastasis using machine learning model

**Fig. 6:**
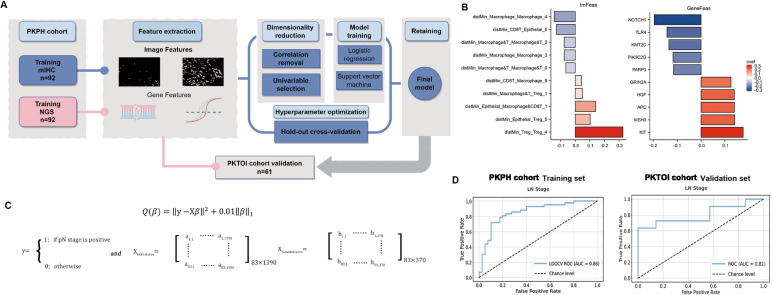
Machine Learning Model of NGS and mIHC Profiles Predicted LN Stage. **A** A schematic illustrated the machine learning-based strategy, which involved feature identification and model optimization. **B** The formula for calculating the coefficients of our ImGene SVM model was presented. **C** A box plot showed the coefficients of genetic and mIHC features used in the model. **D** ROC curves were presented for our LN stage prediction model applied to the PKPH and independent PKTOI cohorts. The AUC was 0.86 for the training set and 0.81 for the validation set. *PKPH* Peking University People’s Hospital, *PKTOI* Peking University People’s Hospital Thoracic Oncology Institution, *ROC* receiver operating characteristic

To investigate the clinical relevance of genoproteomic features in predicting LN metastasis in LUAD, we assessed the combined utility of NGS and mIHC profiles in predicting LN stage with high accuracy. Our findings indicated that the combined NGS and mIHC profiles, collectively termed ImGene, are predictive of LN stage. This prompted us to investigate whether this integrated data could be utilized to predict LN metastasis, independent of surgical options, through a machine learning approach (Fig. 6A and B). Specifically, the genoproteomic features highlighted in Fig. 6B were selected for their predictive strength within the machine learning model. Consequently, the model identified features that were not directly highlighted in prior NGS or mIHC analyses but still contribute to predicting pN status through their interactions, as validated in both the PKPH cohort and the independent PKTOI cohort.

The ImGene model, developed by integrating both NGS and mIHC profiles, demonstrated superior performance over monomodal models based solely on genetic or mIHC data. It achieved an accuracy of 0.82, an F1 score of 0.83, and an area under the curve (AUC) of 0.86 (Supplementary Fig. 6A). This model demonstrated greater accuracy in estimating the probability of N2 stage in lung cancer than both the Peking University (PKU) model, includes variables factors such as age, tumor size, tumor location, and histological subtypes [33] (AUC = 0.77), and the Fudan University model (AUC = 0.67, Supplementary Fig. 5C) for predicting LN stage [34].

In an independent validation set, the PKTOI cohort, the models demonstrated the following performance: the ImGene model achieved an AUC of 0.80, with an accuracy of 0.81 and an F1 score of 0.77 (Fig. 6D, Supplementary Fig. 6A). In contrast, the GeneFeatures monomodal model, which utilizes only NGS profiles, had an AUC of 0.46, while the ImFeatures monomodal model, based solely on mIHC profiles, reached an AUC of 0.74 (Supplementary Fig. 6C). These results underscored the efficacy of our integrated algorithm in analyzing the genoproteomic profiles of primary LUAD tumors to accurately predict LN stage probability.

## Discussion

The identification of genomic alterations and immune cellular distribution patterns in LUAD has been pivotal in advancing targeted therapies [35–37], leading to significantly improved outcomes in metastatic disease [38–40]. However, the molecular heterogeneity of cancer [41, 42], particularly pronounced across diverse populations [43] and influenced by various survival risk factors [44, 45], underscores the necessity for a comprehensive analysis. This involves examining spatial immunogenomic patterns within different populations. Such integrative research is instrumental in gaining a deeper understanding of LN metastasis, especially in East Asia.

Our findings provided valuable insights into the heterogeneity of LUAD tumors with LN metastasis and highlighted the critical role of genetic alterations, cellular metaclusters and function units in modulating the LN infiltration. We observed that pN positive tumors exhibited significantly higher TMB, tumor mutation count, and intra-tumor heterogeneity compared to pN negative tumors. Additionally, our mIHC analysis identified 9 distinct cellular metaclusters within the TIME. We discerned LN stage-related TIME subtypes and functional units composed of these diverse metaclusters. Our research also highlighted distinct CN function units strongly correlated with the frequency of metaclusters, unveiling unique patterns of immune cell infiltration linked to genomic alterations in pN positive tumors relative to pN negative ones. We found that the TIME subtypes associated with pN positive tumors are characterized by a higher frequency of *PIK3CG, ATM, BRD4,* and *KMT2B* mutations, and show increased macrophage frequency alongside reduced epithelial cell infiltration. Furthermore, our study recognized the complex interplay between genetic factors and TIME as a pivotal influence on the process of LN metastasis. Building on the genetic and TIME features, we developed a predictive model. The model effectively estimated the likelihood of LN metastasis in LUAD, marking a significant advancement in our understanding of the metastasis disease.

Our NGS findings emphasized the significance of investigating LUAD in different ethnic populations, as supported by previous research [41, 46, 47]. Prior studies have delved into the preoperative clinical and genomic landscapes correlating with pathologic LN metastasis in early-stage LUAD, particularly stages I and II. These investigations, notably within the MSK cohort [9], elucidated significant associations between *STK11* and *SMARCA4* mutations and LN metastasis. In contrast, our research provided a distinct perspective, revealing new genomic dimensions in our PKPH LUAD cohort with LN metastasis. We observed a conspicuous elevation in the frequency of *TP53* and *PIK3CG* mutations, suggesting these are pivotal genetic alterations in pN positive LUAD tumors. Our results resonated with previous literature that identified an augmented occurrence of *TP53* mutations in advanced LN stage LUAD, indicating the potential implication of *TP53* as a critical factor in LN metastasis, possibly mediated through p53 signaling pathways. Multiple studies have robustly associated *TP53* mutations with the progression of LUAD to metastatic states, particularly affecting LN metastasis [48–50]. Functionally, *TP53* mutations were instrumental in cancer proliferation, primarily by facilitating unrestrained cellular growth and division [51, 52].

Our investigation revealed a significantly higher incidence of *PIK3CG* alterations in pN positive primary tumors within the PKPH cohort. This finding indicated an even greater prevalence of *PIK3CG* alterations than what has been reported in the MSK cohort [53], suggesting a potential variance in the molecular underpinnings of LN metastasis across different ethnicities. Furthermore, mutations and amplifications of *PIK3CG* were commonly detected in a range of malignancies, including melanoma, as well as prostate, uterine, gastric, and lung cancers [54–57]. Notably, in LUAD, the PI3K signaling pathway was significantly associated with an increased risk of metastasis [58]. In line with our findings, mutations in the PI3K-AKT-mTOR pathway, observed in various cancers including LUAD, might be linked to the development of tumors advanced LN stages. These insights provided a robust foundation for future investigations into the role of *PIK3CG* in cancer lymph node metastasis.

Genetic aberrations, in synergy with the TIME, were pivotal determinants in the pathology of LN metastasis. This interplay between genetic modifications and the intricacies of TIME underscored its critical significance. Predominantly expressed in macrophages, *PIK3CG* displayed a marked contrast to its lower expression levels in tumor cells [59]. Investigations in *PIK3CG*-deficient mice showcased an augmented macrophage-mediated inflammatory response when confronted with pathogenic stimuli. Subsequent studies illuminated that tumor cells, activated the PI3K/PKB signaling cascade, culminating in enhanced expression of M2 markers and facilitating M2 macrophage polarization [60]. In congruence with these findings, our analysis delineated a comprehensive impact of *PIK3CG* mutations within the TIME. These mutations not only significantly altered the cellular dynamics of macrophages but also impacted their spatial CNs in both pN positive and pN negative tumor stages. Fundamentally, the extensive influence of *PIK3CG* mutations on the immune milieu of tumors was instrumental in elucidating the complex mechanisms underlying LN metastasis, with a distinct focus on the compositional and spatial attributes within the TIME, predominantly reflected in the activities of macrophages and Tregs.

In our study, we established a significant association of macrophages with LN metastasis, particularly evident through their interactions within spatial cellular metaclusters. Macrophages are crucial in the cancer ecosystem, facilitating communication with tumor cells and instigating inflammation [61–63], which subsequently bolsters tumor growth and invasion [64, 65]. Additionally, their role in neovascularization, or new blood vessel formation, is essential for the metastatic spread of cancer cells, especially to the LN [66, 67]. These findings corroborate the central role of macrophages in the LN metastasis tumor microenvironment, emphasizing a spatial perspective [68]. Another study highlighted the increased expression of IL-1β in macrophages following exposure to particulate matter, indicating its necessity for *EGFR*-driven. In contrast, our research did not consistently align with *EGFR*-driven patterns, likely due to racial and environmental variations. Additionally, we identified that the proportion of Tregs and associated immune-suppressed-enriched CN perform distinct functions in LN metastasis. This aligns with the known role of Tregs in targeting tumor cells [69]. In summary, while prior studies have focused on cellular alterations in isolation, our research offered a comprehensive view of cellular interactions, integrating not only quantitative differences but also spatial and functional analyses.

Additionally, our investigation involved developing machine learning models that combine gene alteration and mIHC data for predicting LN metastasis. The high accuracy achieved in independent external validation underscores the robustness of these models, suggesting their potential in utilizing spatial immunogenomic features to provide personalized staging recommendations for LUAD patients ineligible for surgery.

Overall, our investigation provided detailed insights into the complex relationship between genetic factors and the TIME in influencing LN metastasis in LUAD priamry tumors. Through NGS and mIHC, we identified spatial immunogenomic patterns that clarified genomic alterations, cellular interactions within the TIME. These findings contribute to a better understanding of the variability in LN metastasis and point to new directions for exploring metastasis in LUAD primary tumors.

## Limitation

Our study has several limitations that should be acknowledged. Firstly, the NGS analysis was based on single-region sampling of the primary tumor, which may not fully capture the genetic intratumoral heterogeneity, including clonal architecture, that is inherent to LUAD. To better understand the tumor’s complexity, future studies could employ multiple-region sampling or alternative techniques. Additionally, the absence of RNA sequencing data limited our ability to analyze transcriptional patterns and underlying mechanisms. Integrating RNA sequencing would allow for deeper insights into gene expression profiles and molecular interactions within the TIME. Future research should consider incorporating RNA sequencing to enhance the depth and robustness of the findings. Lastly, external validation is needed to assess the performance and generalizability of our model. Testing its predictive accuracy and reliability in independent cohorts or real-world clinical settings would provide valuable confirmation of its utility.

## Supplementary Information

Additional file 1: Table 1. Preoperative clinicopathologic and genomic features associated with pathologic LN metastasis in PKPH NGS cohort. Table 2.1. Patterns of co-occurrence and mutual exclusivity in the PKPH NGS cohort. Table 2.2. Co-occurrence and mutual exclusivity patterns in the pN positive patients of the PKPH NGS cohort. Table 2.3. Co-occurrence and mutual exclusivity patterns in the pN negative patients of the PKPH NGS cohort. Table 3. Glossary. Table 4. Clinicopathologic characteristics of the PKTOI cohort. Figure 1. Univariable and Multivariable Logistic Regression Analysis. A: Univariable logistic regression analysis was performed on preoperative clinicopathologic and genomic features associated with pathologic LN metastasis in the NGS cohort. B: Multivariable logistic regression analysis was conducted on preoperative clinicopathologic and genomic features associated with pathologic LN metastasis in the NGS cohort. Variables with p < 0.05 were highlighted with notable markers. Figure 2. COME Analysis of Oncogenic Pathways. A-C: Co-occurrence (red) and mutual exclusivity (blue) patterns of driver genes were analyzed in the entire cohort, as well as in pN positive and negative groups. D-F: COME analysis of the mitotic pathway was conducted in our whole cohort, pN negative, and pN positive groups. G-I: COME analysis of the mitotic pathway was performed in the MSK cohort, focusing on pN negative and pN positive groups. Abbreviation: COME, co-occurrence and mutual exclusivity. Figure 3. Cellular Metacluster Densities According to the Cellular Immunologic Distribution in LUAD Primary Tumors (n = 92). A, B: The distribution of the numbers of cellular connections and pairs of cell distances among the cellular metaclusters was analyzed. C, D, E: The cell distances (µm) between diverse cellular metacluster pairs were compared: * p < 0.05, ** p < 0.001. Data were presented as means ± SEMs. Statistical analysis was conducted using the Mann-Whitney U test. Figure 4. Genetic Features Analysis of TIME Patterns. A: The silhouette coefficient of the TIME subtypes was calculated and presented. B: Co-occurrence gene analysis was performed for the TIME subtypes. C: The mutation frequencies of genes were compared among the TIME subtypes. Abbreviation: TIME, tumor immune microenvironment. Figure 5. Identification of the Immunogenomic Features According to CN Distribution in the Primary Tumor. A: The distribution of CN profiles was analyzed across the PKPH mIHC cohort (n = 92). B: Statistical analysis showed a positive correlation between epithelial CNs and the proportion of epithelial metaclusters (p < 0.05). No significant difference was observed in the epithelial-enriched CNs (Epithelial-CN 2, 5, 6) between pN negative and positive primary tumor groups (p > 0.05). C: Statistical analysis revealed a positive correlation between CNs enriched in CD8+ and CD4+ T cells (CN 1) and the proportion of T cell metaclusters (p < 0.05). No significant difference was observed in CN 1 between pN negative and positive primary tumor groups (p > 0.05). D: Immunogenomic patterns were integrated with genetic alterations and CNs in our PKPH cohort. Abbreviations: CN, cellular neighborhoods; PKPH, Peking University People’s Hospital. Figure 6. ImGene Model Predicted the LN Stage. A, B, C: The assessment indices of the ImGene multimodel, using genetic and mIHC data, exhibited superior performance compared to the ImFeatures monomodels on both the training and validation sets. D: The ROC curve of the PKU and FUDAN N2 stage prediction model was presented. Abbreviations: PKPH, Peking University People’s Hospital; PKTOI, Peking University People’s Hospital Thoracic Oncology Institution; ROC, receiver operating characteristic; PKU, Peking University.

## Data Availability

No datasets were generated or analysed during the current study.
