# Supplementary material for: Spatial immunogenomic patterns associated with lymph node metastasis in lung adenocarcinoma
Source: Exp Hematol Oncol. 2024 Oct 28;13:106. doi: 10.1186/s40164-024-00574-8 (PMC11514955; doi:10.1186/s40164-024-00574-8)
Supplement: Supplementary file 1 — Additional file 1: Table 1. Preoperative clinicopathologic and genomic features associated with pathologic LN metastasis in PKPH NGS cohort. Table 2.1. Patterns of co-occurrence and mutual exclusivity in the PKPH NGS cohort. Table 2.2. Co-occurrence and mutual exclusivity patterns in the pN positive patients of the PKPH NGS cohort. Table 2.3. Co-occurrence and mutual exclusivity patterns in the pN negative patients of the PKPH NGS cohort. Table 3. Glossary. Table 4. Clinicopathologic characteristics of the PKTOI cohort. Figure 1. Univariable and Multivariable Logistic Regression Analysis. A: Univariable logistic regression analysis was performed on preoperative clinicopathologic and genomic features associated with pathologic LN metastasis in the NGS cohort. B: Multivariable logistic regression analysis was conducted on preoperative clinicopathologic and genomic features associated with pathologic LN metastasis in the NGS cohort. Variables with p < 0.05 were highlighted with notable markers. Figure 2. COME Analysis of Oncogenic Pathways. A-C: Co-occurrence (red) and mutual exclusivity (blue) patterns of driver genes were analyzed in the entire cohort, as well as in pN positive and negative groups. D-F: COME analysis of the mitotic pathway was conducted in our whole cohort, pN negative, and pN positive groups. G-I: COME analysis of the mitotic pathway was performed in the MSK cohort, focusing on pN negative and pN positive groups. Abbreviation: COME, co-occurrence and mutual exclusivity. Figure 3. Cellular Metacluster Densities According to the Cellular Immunologic Distribution in LUAD Primary Tumors (n = 92). A, B: The distribution of the numbers of cellular connections and pairs of cell distances among the cellular metaclusters was analyzed. C, D, E: The cell distances (µm) between diverse cellular metacluster pairs were compared: * p < 0.05, ** p < 0.001. Data were presented as means ± SEMs. Statistical analysis was conducted using the Mann-Whitney U test. Figure [file 40164_2024_574_MOESM1_ESM.zip › New folder/40164_2024_574_MOESM1_ESM.docx]

**Supplementary Table 2.1 Patterns of co-occurrence and mutual exclusivity in the PKPH NGS cohort**

| **Gene1** | **Gene2** | **p value** | **Event** | **Event_ratio** |
| --- | --- | --- | --- | --- |
| *KRAS* | *EGFR* | 1.0668106790366e-13 | Mutually Exclusive | 2/198 |
| *KRAS* | *STK11* | 8.02343464960901e-08 | Co-Occurence | 9/24 |
| *STK11* | *EGFR* | 3.7554316845796e-06 | Mutually Exclusive | 0/182 |
| *KEAP1* | *STK11* | 2.66565996823814e-05 | Co-Occurence | 4/9 |
| *EGFR* | *BRAF* | 9.67023658779242e-05 | Mutually Exclusive | 1/180 |
| *KEAP1* | *EGFR* | 0.00124596424298776 | Mutually Exclusive | 0/177 |
| *KRAS* | *KEAP1* | 0.00222379965901065 | Co-Occurence | 4/29 |
| *RBM10* | *EGFR* | 0.00565540811316006 | Co-Occurence | 26/148 |
| *SETD2* | *BRAF* | 0.00803359294651465 | Co-Occurence | 3/16 |
| *KRAS* | *HGF* | 0.0086844428788462 | Co-Occurence | 4/31 |
| *ARID1A* | *RAF* | 0.0104554517381997 | Co-Occurence | 3/17 |
| *ARID1A* | *TP53* | 0.0172117441986136 | Co-Occurence | 9/99 |
| *ARID1A* | *PTEN* | 0.01853110808042 | Co-Occurence | 2/13 |
| *KEAP1* | *SETD2* | 0.0228010644999404 | Co-Occurence | 2/13 |
| *STK11* | *HGF* | 0.0405840890567363 | Co-Occurence | 2/15 |
| *KRAS* | *ARID1A* | 0.0430605503046941 | Co-Occurence | 4/35 |
| *HGF* | *ARID1A* | 0.0479309998886531 | Co-Occurence | 2/16 |
| *TP53* | *HGF* | 0.0663796394650639 | Co-Occurence | 6/101 |
| *RB1* | *TP53* | 0.0962739578229447 | Co-Occurence | 7/101 |
| *SF3B1* | *EGFR* | 0.0988262370291754 | Mutually Exclusive | 2/173 |
| *EGFR* | *ARID1A* | 0.112165381920841 | Mutually Exclusive | 5/173 |
| *EGFR* | *RB1* | 0.171809567924061 | Co-Occurence | 9/163 |
| *PTEN* | *STK11* | 0.197869277700277 | Co-Occurence | 1/14 |
| *TP53* | *SF3B1* | 0.229618919130359 | Co-Occurence | 4/103 |
| *BRAF* | *KEAP1* | 0.232882920340344 | Co-Occurence | 1/15 |
| *SETD2* | *SF3B1* | 0.232882920340344 | Co-Occurence | 1/15 |
| *STK11* | *SF3B1* | 0.232882920340344 | Co-Occurence | 1/15 |
| *BRAF* | *HGF* | 0.298775526864098 | Co-Occurence | 1/17 |
| *RBM10* | *RB1* | 0.313947346544761 | Co-Occurence | 2/35 |
| *RB1* | *BRAF* | 0.359481998450193 | Co-Occurence | 1/19 |
| *BRAF* | *TP53* | 0.364388310670744 | Co-Occurence | 6/104 |
| *SETD2* | *TP53* | 0.364388310670744 | Co-Occurence | 6/104 |
| *STK11* | *BRAF* | 0.388007091636621 | Co-Occurence | 1/20 |
| *STK11* | *SETD2* | 0.388007091636621 | Co-Occurence | 1/20 |
| PKPH, Peking University People’s Hospital; NGS, next-generation sequencing. | | | | |

**Supplementary Table 2.2 Co-occurrence and mutual exclusivity patterns in the pN positive patients of the PKPH NGS cohort**

| **Gene1** | **Gene2** | **p value** | **Event** | **Event_ratio** |
| --- | --- | --- | --- | --- |
| *EGFR* | *STK11* | 0.0117256772084741 | Mutually Exclusive | 0/35 |
| *STK11* | *KRAS* | 0.013191691771048 | Co-Occurence | 3/6 |
| *STK11* | *KEAP1* | 0.020916929906941 | Co-Occurence | 2/4 |
| *EGFR* | *BRAF* | 0.030239904379749 | Mutually Exclusive | 0/34 |
| *KRAS* | *EGFR* | 0.0344444071053984 | Mutually Exclusive | 1/35 |
| *KRAS* | *KEAP1* | 0.0427302996670366 | Co-Occurence | 2/6 |
| *PTEN* | *ARID1A* | 0.0747460087082727 | Co-Occurence | 1/2 |
| *EGFR* | *KEAP1* | 0.0755997609493725 | Mutually Exclusive | 0/33 |
| *HGF* | *KRAS* | 0.0798429095876377 | Co-Occurence | 2/7 |
| *TP53* | *KEAP1* | 0.0863997267992828 | Mutually Exclusive | 0/32 |
| *BRAF* | *ARID1A* | 0.146589259796807 | Co-Occurence | 1/4 |
| *SETD2* | *ARID1A* | 0.146589259796807 | Co-Occurence | 1/4 |
| *SF3B1* | *SETD2* | 0.146589259796807 | Co-Occurence | 1/4 |
| *TP53* | *EGFR* | 0.174459727155953 | Co-Occurence | 19/21 |
| *STK11* | *SF3B1* | 0.181422351233672 | Co-Occurence | 1/5 |
| *EGFR* | *SF3B1* | 0.183599419448476 | Mutually Exclusive | 0/32 |
| *SETD2* | *KEAP1* | 0.213523435499018 | Co-Occurence | 1/5 |
| *HGF* | *RBM10* | 0.213523435499018 | Co-Occurence | 1/5 |
| *RB1* | *RBM10* | 0.213523435499018 | Co-Occurence | 1/5 |
| *RBM10* | *EGFR* | 0.248911465892598 | Co-Occurence | 3/27 |
| *RB1* | *BRAF* | 0.276441560659097 | Co-Occurence | 1/6 |
| *SETD2* | *BRAF* | 0.276441560659097 | Co-Occurence | 1/6 |
| *EGFR* | *SETD2* | 0.305267651327585 | Mutually Exclusive | 1/32 |
| *STK11* | *HGF* | 0.335507555707334 | Co-Occurence | 1/7 |
| *STK11* | *SETD2* | 0.335507555707334 | Co-Occurence | 1/7 |
| *KRAS* | *RBM10* | 0.352002049005379 | Co-Occurence | 1/8 |
| *SETD2* | *KRAS* | 0.442721762144626 | Co-Occurence | 1/9 |
| *TP53* | *PTEN* | 0.494920174165457 | Co-Occurence | 2/27 |
| *EGFR* | *PTEN* | 0.499274310595065 | Co-Occurence | 2/28 |
| *HGF* | *TP53* | 0.617327755485358 | Co-Occurence | 3/27 |
| *RB1* | *TP53* | 0.617327755485358 | Co-Occurence | 3/27 |
| *RB1* | *EGFR* | 0.62416118842312 | Co-Occurence | 3/28 |
| *TP53* | *STK11* | 0.648566306057982 | Mutually Exclusive | 2/30 |
| *ARID1A* | *KEAP1* | 1 | Mutually Exclusive | 0/5 |

**Supplementary Table 2.3 Co-occurrence and mutual exclusivity patterns in the pN negative patients of the PKPH NGS cohort**

| **Gene1** | **Gene2** | **p value** | **Event** | **Event_ratio** |
| --- | --- | --- | --- | --- |
| *KRAS* | *EGFR* | 1.26047653057953e-12 | Mutually Exclusive | 1/163 |
| *STK11* | *KRAS* | 1.44813131705394e-06 | Co-Occurence | 6/18 |
| *STK11* | *EGFR* | 0.000731032399281152 | Mutually Exclusive | 0/147 |
| *STK11* | *KEAP1* | 0.00214459290032161 | Co-Occurence | 2/5 |
| *BRAF* | *EGFR* | 0.00385453446893698 | Mutually Exclusive | 1/146 |
| *ARID1A* | *TP53* | 0.00627262972439687 | Co-Occurence | 8/70 |
| *HGF* | *ARID1A* | 0.0123601261461256 | Co-Occurence | 2/10 |
| *ARID1A* | *KRAS* | 0.0193051582655309 | Co-Occurence | 4/26 |
| *SETD2* | *BRAF* | 0.0195927284589113 | Co-Occurence | 2/10 |
| *EGFR* | *RBM10* | 0.0230055430949596 | Co-Occurence | 23/121 |
| *KEAP1* | *EGFR* | 0.0284829192858434 | Mutually Exclusive | 0/144 |
| *KEAP1* | *KRAS* | 0.0370849603070999 | Co-Occurence | 2/23 |
| *ARID1A* | *BRAF* | 0.0399212629556322 | Co-Occurence | 2/13 |
| *HGF* | *KRAS* | 0.0688180410181734 | Co-Occurence | 2/24 |
| *EGFR* | *ARID1A* | 0.0721701951628866 | Mutually Exclusive | 4/143 |
| *PTEN* | *STK11* | 0.0860763561143133 | Co-Occurence | 1/7 |
| *KEAP1* | *BRAF* | 0.0999236840519752 | Co-Occurence | 1/8 |
| *SETD2* | *KEAP1* | 0.0999236840519752 | Co-Occurence | 1/8 |
| *HGF* | *STK11* | 0.113357658916871 | Co-Occurence | 1/8 |
| *BRAF* | *HGF* | 0.131269625403399 | Co-Occurence | 1/9 |
| *ARID1A* | *PTEN* | 0.140625044828447 | Co-Occurence | 1/11 |
| *HGF* | *TP53* | 0.146753615404876 | Co-Occurence | 3/74 |
| *SF3B1* | *TP53* | 0.146753615404876 | Co-Occurence | 3/74 |
| *RB1* | *EGFR* | 0.180074094315154 | Co-Occurence | 6/135 |
| *BRAF* | *STK11* | 0.191164000978602 | Co-Occurence | 1/11 |
| *KRAS* | *BRAF* | 0.192999631991784 | Co-Occurence | 2/27 |
| *RB1* | *TP53* | 0.198344266842768 | Co-Occurence | 4/74 |
| *PTEN* | *EGFR* | 0.225995621874561 | Mutually Exclusive | 1/142 |
| *ARID1A* | *STK11* | 0.263195387849671 | Co-Occurence | 1/14 |
| *PTEN* | *KRAS* | 0.314404491738655 | Co-Occurence | 1/25 |
| *RBM10* | *KRAS* | 0.324565985091705 | Mutually Exclusive | 1/48 |
| *SF3B1* | *RBM10* | 0.42287438153158 | Co-Occurence | 1/28 |
| *TP53* | *BRAF* | 0.427930024123352 | Co-Occurence | 4/75 |
| *TP53* | *SETD2* | 0.427930024123352 | Co-Occurence | 4/75 |

**Supplementary Table 3 Glossary**

| **Abbreviations** | **Full Name** |
| --- | --- |
| LUAD  LN  NGS  CNVs  MATH  ITH  VAF  TIME  mIHC  CNs  TMB  COME  ACI  LEP  MIP  LVI  PAP  SOL  STAS  VPI  OncoKB  Tregs  MF  AUC  ROC  MIA  AJCC  FDR  pN+  pN0  TSG  NPA  SEM  PKU | Lung adenocarcinoma  lymph node  next-generation sequencing  copy number variations  mutant-allele tumor heterogeneity  intratumor heterogeneity  variant allele frequency  tumor immune microenvironment  multiplex Immunohistochemistry  cellular neighbourhoods  tumor mutational burden  co-occurrence and mutual exclusivity  acinar  lepidic  micropapillary  lymphovascular invasion  papillary  solid  spread through air spaces  visceral pleural invasion  oncology knowledge base  T regulatory cells  macrophage  area under curve  receiver operating characteristic curve  minimally invasive adenocarcinoma  American Joint Committee on Cancer  false discovery rate  pN positive  pN negative  tumor suppressor gene  number of pathway alteration  standard error of mean  Peking University |

**Supplementary Table 4 Clinicopathologic characteristics of the PKTOI cohort**

| **Characteristics** | **pN negative** | **pN positive** | **p value** |
| --- | --- | --- | --- |
| n | 26 | 35 |  |
| Age, median (IQR) | 61.2 ± 11.1 | 58.1 ± 12.7 | 0.318 |
| Sex, n (%) |  |  | 0.798 |
| Male | 12 (19.7%) | 15 (24.6%) |  |
| Female | 14 (23%) | 20 (32.8%) |  |
| Smoking, n (%) |  |  | 0.334 |
| No | 8 (13.1%) | 7 (11.5%) |  |
| Yes | 18 (29.5%) | 28 (45.9%) |  |
| Nodule_type, n (%) |  |  | < 0.001 |
| solid | 23 (37.7%) | 11 (18%) |  |
| subsolid | 3 (4.9%) | 24 (39.3%) |  |
| Path_STAS, n (%) |  |  | 0.218 |
| no | 21 (34.4%) | 33 (54.1%) |  |
| yes | 5 (8.2%) | 2 (3.3%) |  |
| Path_VPI, n (%) |  |  | 0.956 |
| no | 18 (29.5%) | 24 (39.3%) |  |
| yes | 8 (13.1%) | 11 (18%) |  |
| Path_LVI, n (%) |  |  | 0.426 |
| no | 25 (41%) | 35 (57.4%) |  |
| yes | 1 (1.6%) | 0 (0%) |  |
| Stage, n (%) |  |  | < 0.001 |
| ⅠA2 | 0 (0%) | 13 (21.3%) |  |
| ⅠA3 | 0 (0%) | 12 (19.7%) |  |
| ⅠB | 0 (0%) | 9 (14.8%) |  |
| ⅡB | 7 (11.5%) | 0 (0%) |  |
| ⅢA | 18 (29.5%) | 0 (0%) |  |
| ⅢB | 1 (1.6%) | 0 (0%) |  |
| Note: Data are number (%) or median (interquartile range).  PKTOI, the Peking University People’s Hospital Thoracic Oncology Institution;  SOL, solid; STAS, spread through air spaces; VPI, visceral pleural invasion. | | | |

**Supplementary Figure 1 Univariable and Multivariable Logistic Regression Analysis.** A: Univariable logistic regression analysis was performed on preoperative clinicopathologic and genomic features associated with pathologic LN metastasis in the NGS cohort. B: Multivariable logistic regression analysis was conducted on preoperative clinicopathologic and genomic features associated with pathologic LN metastasis in the NGS cohort. Variables with p < 0.05 were highlighted with notable markers.

**Supplementary Figure 2 COME Analysis of Oncogenic Pathways.** A-C: Co-occurrence (red) and mutual exclusivity (blue) patterns of driver genes were analyzed in the entire cohort, as well as in pN positive and negative groups. D-F: COME analysis of the mitotic pathway was conducted in our whole cohort, pN negative, and pN positive groups. G-I: COME analysis of the mitotic pathway was performed in the MSK cohort, focusing on pN negative and pN positive groups. Abbreviation: COME, co-occurrence and mutual exclusivity.

**Supplementary Figure 3 Cellular Metacluster Densities According to the Cellular Immunologic Distribution in LUAD Primary Tumors (n = 92).** A, B: The distribution of the numbers of cellular connections and pairs of cell distances among the cellular metaclusters was analyzed. C, D, E: The cell distances (µm) between diverse cellular metacluster pairs were compared: * p < 0.05, ** p < 0.001. Data were presented as means ± SEMs. Statistical analysis was conducted using the Mann-Whitney U test.

**Supplementary Figure 4 Genetic Features Analysis of TIME Patterns.** A: The silhouette coefficient of the TIME subtypes was calculated and presented. B: Co-occurrence gene analysis was performed for the TIME subtypes. C: The mutation frequencies of genes were compared among the TIME subtypes. Abbreviation: TIME, tumor immune microenvironment.

**Supplementary Figure 5 Identification of the Immunogenomic Features According to CN Distribution in the Primary Tumor.** A: The distribution of CN profiles was analyzed across the PKPH mIHC cohort (n = 92). B: Statistical analysis showed a positive correlation between epithelial CNs and the proportion of epithelial metaclusters (p < 0.05). No significant difference was observed in the epithelial-enriched CNs (Epithelial-CN 2, 5, 6) between pN negative and positive primary tumor groups (p > 0.05). C: Statistical analysis revealed a positive correlation between CNs enriched in CD8^+^ and CD4^+^ T cells (CN 1) and the proportion of T cell metaclusters (p < 0.05). No significant difference was observed in CN 1 between pN negative and positive primary tumor groups (p > 0.05). D: Immunogenomic patterns were integrated with genetic alterations and CNs in our PKPH cohort. Abbreviations: CN, cellular neighborhoods; PKPH, Peking University People’s Hospital.

**Supplementary Figure 6 ImGene Model Predicted the LN Stage**. A, B, C: The assessment indices of the ImGene multimodel, using genetic and mIHC data, exhibited superior performance compared to the ImFeatures monomodels on both the training and validation sets. D: The ROC curve of the PKU and FUDAN N2 stage prediction model was presented. Abbreviations: PKPH, Peking University People’s Hospital; PKTOI, Peking University People’s Hospital Thoracic Oncology Institution; ROC, receiver operating characteristic; PKU, Peking University.
