# Supplementary material for: Spatial immunogenomic patterns associated with lymph node metastasis in lung adenocarcinoma
Source: Exp Hematol Oncol. 2024 Oct 28;13:106. doi: 10.1186/s40164-024-00574-8 (PMC11514955; doi:10.1186/s40164-024-00574-8)
Supplement: Supplementary file 1 — Additional file 1: Table 1. Preoperative clinicopathologic and genomic features associated with pathologic LN metastasis in PKPH NGS cohort. Table 2.1. Patterns of co-occurrence and mutual exclusivity in the PKPH NGS cohort. Table 2.2. Co-occurrence and mutual exclusivity patterns in the pN positive patients of the PKPH NGS cohort. Table 2.3. Co-occurrence and mutual exclusivity patterns in the pN negative patients of the PKPH NGS cohort. Table 3. Glossary. Table 4. Clinicopathologic characteristics of the PKTOI cohort. Figure 1. Univariable and Multivariable Logistic Regression Analysis. A: Univariable logistic regression analysis was performed on preoperative clinicopathologic and genomic features associated with pathologic LN metastasis in the NGS cohort. B: Multivariable logistic regression analysis was conducted on preoperative clinicopathologic and genomic features associated with pathologic LN metastasis in the NGS cohort. Variables with p < 0.05 were highlighted with notable markers. Figure 2. COME Analysis of Oncogenic Pathways. A-C: Co-occurrence (red) and mutual exclusivity (blue) patterns of driver genes were analyzed in the entire cohort, as well as in pN positive and negative groups. D-F: COME analysis of the mitotic pathway was conducted in our whole cohort, pN negative, and pN positive groups. G-I: COME analysis of the mitotic pathway was performed in the MSK cohort, focusing on pN negative and pN positive groups. Abbreviation: COME, co-occurrence and mutual exclusivity. Figure 3. Cellular Metacluster Densities According to the Cellular Immunologic Distribution in LUAD Primary Tumors (n = 92). A, B: The distribution of the numbers of cellular connections and pairs of cell distances among the cellular metaclusters was analyzed. C, D, E: The cell distances (µm) between diverse cellular metacluster pairs were compared: * p < 0.05, ** p < 0.001. Data were presented as means ± SEMs. Statistical analysis was conducted using the Mann-Whitney U test. Figure [file 40164_2024_574_MOESM1_ESM.zip › New folder/Supplementary Figure 1.pdf]

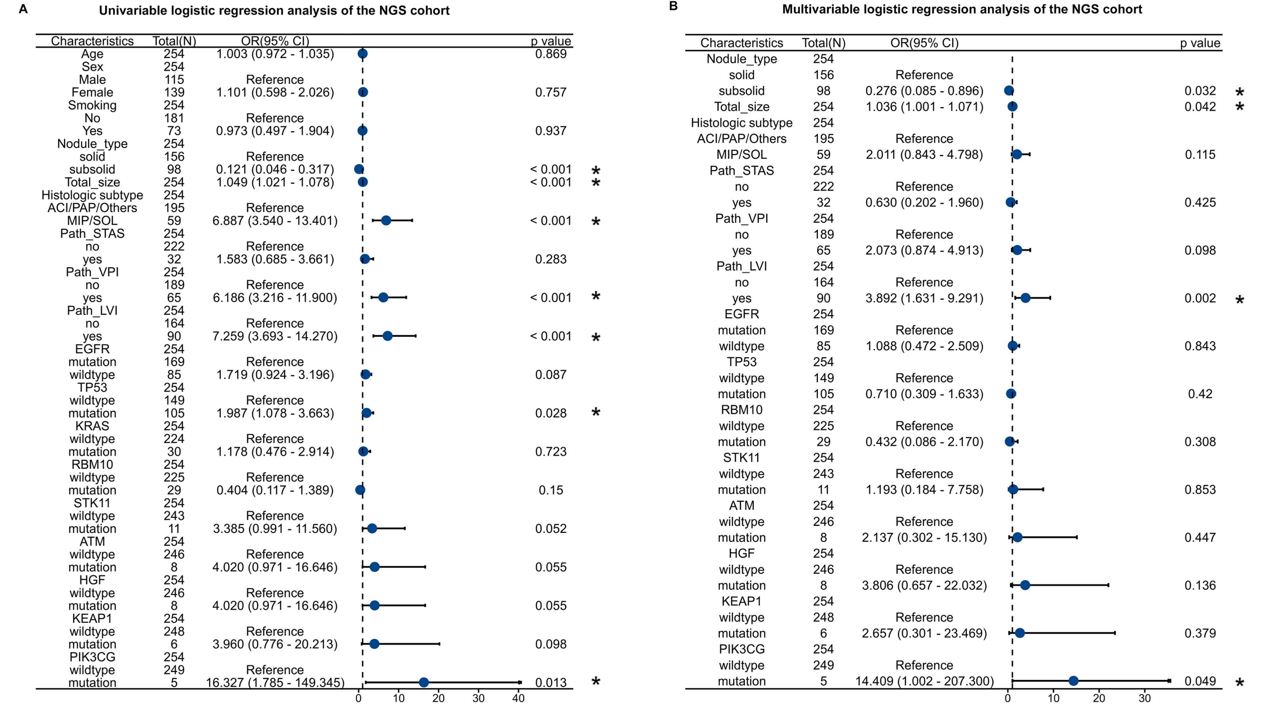
A
Univariable logistic regression analysis of the NGS cohort
Characteristics Total(N) OR(95% CI) p value
Age 254 1.003 (0.972 - 1.035) 0.869
Sex 254
Male 115 Reference
Female 139 1.101 (0.598 - 2.026) 0.757
Smoking 254
No 181 Reference
Yes 73 0.973 (0.497 - 1.904) 0.937
Nodule_type 254
solid 156 Reference
subsolid 98 0.121 (0.046 - 0.317) < 0.001 *
Total_size 254 1.049 (1.021 - 1.078) < 0.001 *
Histologic subtype 254
ACI/PAP/Others 195 Reference
MIP/SOL 59 6.887 (3.540 - 13.401) < 0.001 *
Path_STAS 254
no 222 Reference
yes 32 1.583 (0.685 - 3.661) 0.283
Path_VPI 254
no 189 Reference
yes 65 6.186 (3.216 - 11.900) < 0.001 *
Path_LVI 254
no 164 Reference
yes 90 7.259 (3.693 - 14.270) < 0.001 *
EGFR 254
mutation 169 Reference
wildtype 85 1.719 (0.924 - 3.196) 0.087
TP53 254
wildtype 149 Reference
mutation 105 1.987 (1.078 - 3.663) 0.028 *
KRAS 254
wildtype 224 Reference
mutation 30 1.178 (0.476 - 2.914) 0.723
RBM10 254
wildtype 225 Reference
mutation 29 0.404 (0.117 - 1.389) 0.15
STK11 254
wildtype 243 Reference
mutation 11 3.385 (0.991 - 11.560) 0.052
ATM 254
wildtype 246 Reference
mutation 8 4.020 (0.971 - 16.646) 0.055
HGF 254
wildtype 246 Reference
mutation 8 4.020 (0.971 - 16.646) 0.055
KEAP1 254
wildtype 248 Reference
mutation 6 3.960 (0.776 - 20.213) 0.098
PIK3CG 254
wildtype 249 Reference
mutation 5 16.327 (1.785 - 149.345) 0.013 *
0 10 20 30 40
B
Multivariable logistic regression analysis of the NGS cohort
Characteristics Total(N) OR(95% CI) p value
Nodule_type 254
solid 156 Reference
subsolid 98 0.276 (0.085 - 0.896) 0.032 *
Total_size 254 1.036 (1.001 - 1.071) 0.042 *
Histologic subtype 254
ACI/PAP/Others 195 Reference
MIP/SOL 59 2.011 (0.843 - 4.798) 0.115
Path_STAS 254
no 222 Reference
yes 32 0.630 (0.202 - 1.960) 0.425
Path_VPI 254
no 189 Reference
yes 65 2.073 (0.874 - 4.913) 0.098
Path_LVI 254
no 164 Reference
yes 90 3.892 (1.631 - 9.291) 0.002 *
EGFR 254
mutation 169 Reference
wildtype 85 1.088 (0.472 - 2.509) 0.843
TP53 254
wildtype 149 Reference
mutation 105 0.710 (0.309 - 1.633) 0.42
RBM10 254
wildtype 225 Reference
mutation 29 0.432 (0.086 - 2.170) 0.308
STK11 254
wildtype 243 Reference
mutation 11 1.193 (0.184 - 7.758) 0.853
ATM 254
wildtype 246 Reference
mutation 8 2.137 (0.302 - 15.130) 0.447
HGF 254
wildtype 246 Reference
mutation 8 3.806 (0.657 - 22.032) 0.136
KEAP1 254
wildtype 248 Reference
mutation 6 2.657 (0.301 - 23.469) 0.379
PIK3CG 254
wildtype 249 Reference
mutation 5 14.409 (1.002 - 207.300) 0.049 *
0 10 20 30
