# Supplementary figures and images for: Spatial immunogenomic patterns associated with lymph node metastasis in lung adenocarcinoma
Source: Exp Hematol Oncol. 2024 Oct 28;13:106. doi: 10.1186/s40164-024-00574-8 (PMC11514955; doi:10.1186/s40164-024-00574-8)

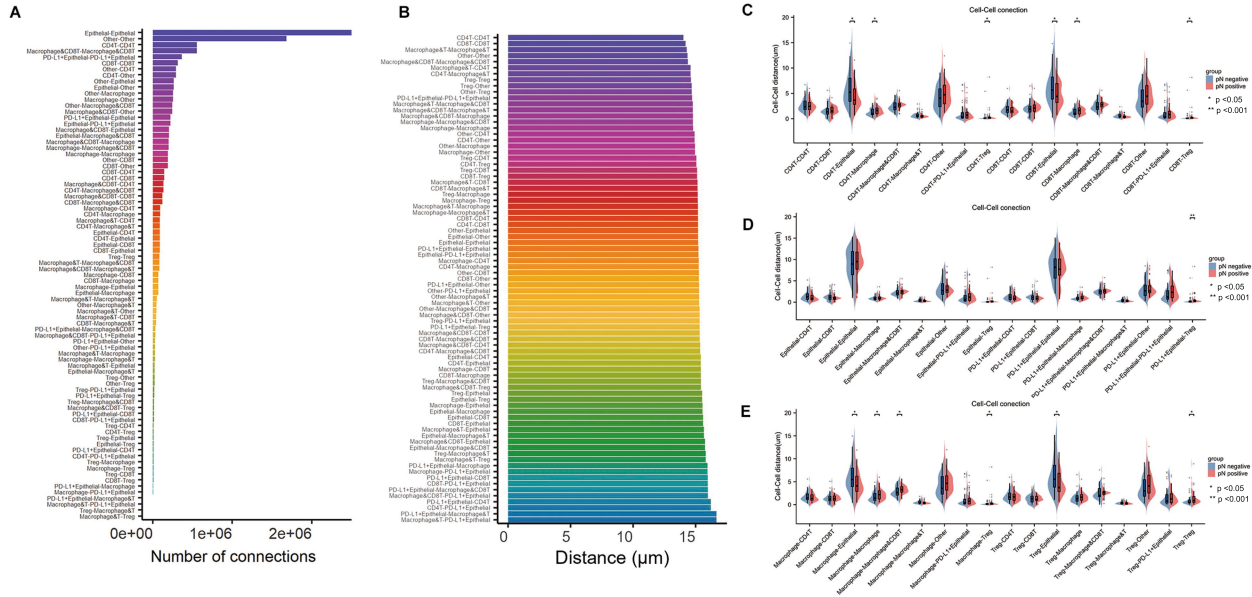

Supplement: Supplementary file 1 — Additional file 1: Table 1. Preoperative clinicopathologic and genomic features associated with pathologic LN metastasis in PKPH NGS cohort. Table 2.1. Patterns of co-occurrence and mutual exclusivity in the PKPH NGS cohort. Table 2.2. Co-occurrence and mutual exclusivity patterns in the pN positive patients of the PKPH NGS cohort. Table 2.3. Co-occurrence and mutual exclusivity patterns in the pN negative patients of the PKPH NGS cohort. Table 3. Glossary. Table 4. Clinicopathologic characteristics of the PKTOI cohort. Figure 1. Univariable and Multivariable Logistic Regression Analysis. A: Univariable logistic regression analysis was performed on preoperative clinicopathologic and genomic features associated with pathologic LN metastasis in the NGS cohort. B: Multivariable logistic regression analysis was conducted on preoperative clinicopathologic and genomic features associated with pathologic LN metastasis in the NGS cohort. Variables with p < 0.05 were highlighted with notable markers. Figure 2. COME Analysis of Oncogenic Pathways. A-C: Co-occurrence (red) and mutual exclusivity (blue) patterns of driver genes were analyzed in the entire cohort, as well as in pN positive and negative groups. D-F: COME analysis of the mitotic pathway was conducted in our whole cohort, pN negative, and pN positive groups. G-I: COME analysis of the mitotic pathway was performed in the MSK cohort, focusing on pN negative and pN positive groups. Abbreviation: COME, co-occurrence and mutual exclusivity. Figure 3. Cellular Metacluster Densities According to the Cellular Immunologic Distribution in LUAD Primary Tumors (n = 92). A, B: The distribution of the numbers of cellular connections and pairs of cell distances among the cellular metaclusters was analyzed. C, D, E: The cell distances (µm) between diverse cellular metacluster pairs were compared: * p < 0.05, ** p < 0.001. Data were presented as means ± SEMs. Statistical analysis was conducted using the Mann-Whitney U test. Figure [file 40164_2024_574_MOESM1_ESM.zip › New folder/Supplementary Figure 3.pdf]

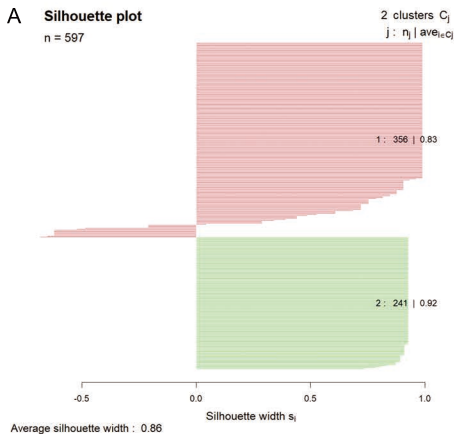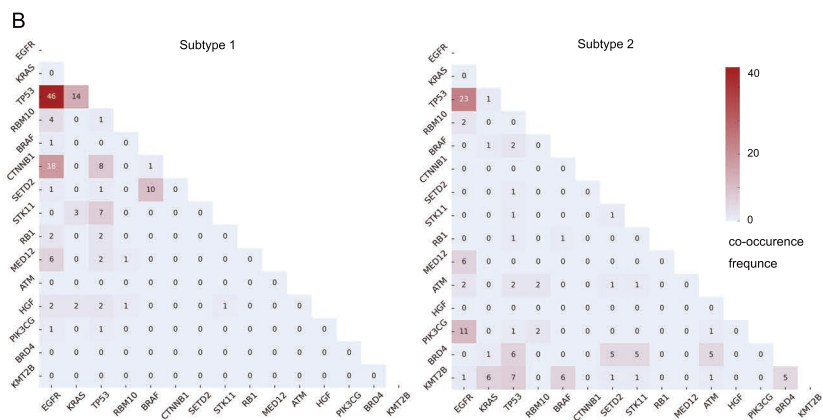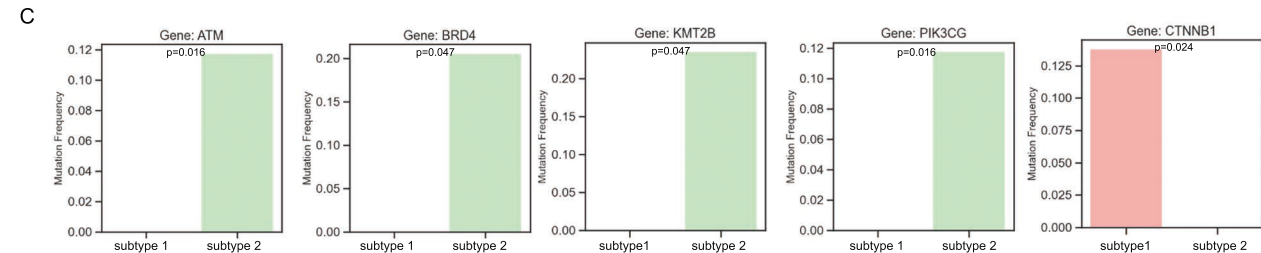

Supplement: Supplementary file 1 — Additional file 1: Table 1. Preoperative clinicopathologic and genomic features associated with pathologic LN metastasis in PKPH NGS cohort. Table 2.1. Patterns of co-occurrence and mutual exclusivity in the PKPH NGS cohort. Table 2.2. Co-occurrence and mutual exclusivity patterns in the pN positive patients of the PKPH NGS cohort. Table 2.3. Co-occurrence and mutual exclusivity patterns in the pN negative patients of the PKPH NGS cohort. Table 3. Glossary. Table 4. Clinicopathologic characteristics of the PKTOI cohort. Figure 1. Univariable and Multivariable Logistic Regression Analysis. A: Univariable logistic regression analysis was performed on preoperative clinicopathologic and genomic features associated with pathologic LN metastasis in the NGS cohort. B: Multivariable logistic regression analysis was conducted on preoperative clinicopathologic and genomic features associated with pathologic LN metastasis in the NGS cohort. Variables with p < 0.05 were highlighted with notable markers. Figure 2. COME Analysis of Oncogenic Pathways. A-C: Co-occurrence (red) and mutual exclusivity (blue) patterns of driver genes were analyzed in the entire cohort, as well as in pN positive and negative groups. D-F: COME analysis of the mitotic pathway was conducted in our whole cohort, pN negative, and pN positive groups. G-I: COME analysis of the mitotic pathway was performed in the MSK cohort, focusing on pN negative and pN positive groups. Abbreviation: COME, co-occurrence and mutual exclusivity. Figure 3. Cellular Metacluster Densities According to the Cellular Immunologic Distribution in LUAD Primary Tumors (n = 92). A, B: The distribution of the numbers of cellular connections and pairs of cell distances among the cellular metaclusters was analyzed. C, D, E: The cell distances (µm) between diverse cellular metacluster pairs were compared: * p < 0.05, ** p < 0.001. Data were presented as means ± SEMs. Statistical analysis was conducted using the Mann-Whitney U test. Figure [file 40164_2024_574_MOESM1_ESM.zip › New folder/Supplementary Figure 4.pdf]

**A**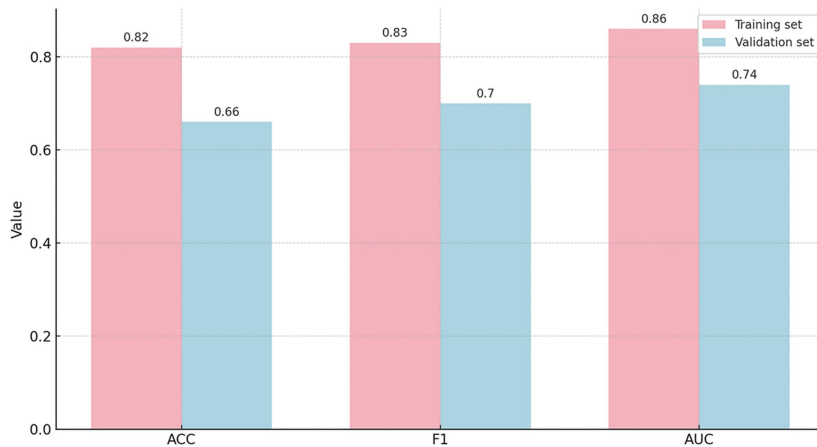**B**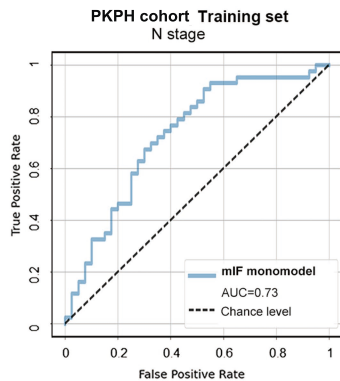**C**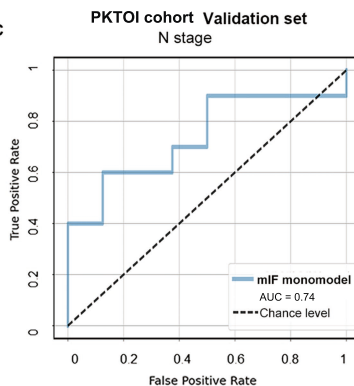**D**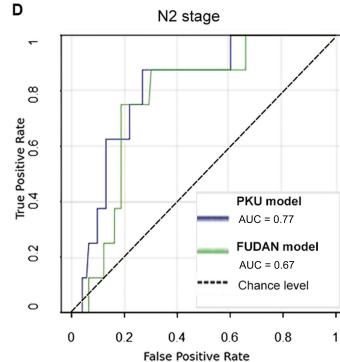

Supplement: Supplementary file 1 — Additional file 1: Table 1. Preoperative clinicopathologic and genomic features associated with pathologic LN metastasis in PKPH NGS cohort. Table 2.1. Patterns of co-occurrence and mutual exclusivity in the PKPH NGS cohort. Table 2.2. Co-occurrence and mutual exclusivity patterns in the pN positive patients of the PKPH NGS cohort. Table 2.3. Co-occurrence and mutual exclusivity patterns in the pN negative patients of the PKPH NGS cohort. Table 3. Glossary. Table 4. Clinicopathologic characteristics of the PKTOI cohort. Figure 1. Univariable and Multivariable Logistic Regression Analysis. A: Univariable logistic regression analysis was performed on preoperative clinicopathologic and genomic features associated with pathologic LN metastasis in the NGS cohort. B: Multivariable logistic regression analysis was conducted on preoperative clinicopathologic and genomic features associated with pathologic LN metastasis in the NGS cohort. Variables with p < 0.05 were highlighted with notable markers. Figure 2. COME Analysis of Oncogenic Pathways. A-C: Co-occurrence (red) and mutual exclusivity (blue) patterns of driver genes were analyzed in the entire cohort, as well as in pN positive and negative groups. D-F: COME analysis of the mitotic pathway was conducted in our whole cohort, pN negative, and pN positive groups. G-I: COME analysis of the mitotic pathway was performed in the MSK cohort, focusing on pN negative and pN positive groups. Abbreviation: COME, co-occurrence and mutual exclusivity. Figure 3. Cellular Metacluster Densities According to the Cellular Immunologic Distribution in LUAD Primary Tumors (n = 92). A, B: The distribution of the numbers of cellular connections and pairs of cell distances among the cellular metaclusters was analyzed. C, D, E: The cell distances (µm) between diverse cellular metacluster pairs were compared: * p < 0.05, ** p < 0.001. Data were presented as means ± SEMs. Statistical analysis was conducted using the Mann-Whitney U test. Figure [file 40164_2024_574_MOESM1_ESM.zip › New folder/Supplementary Figure 6 .pdf]
